# Transcriptional signatures of the whole-brain voxel-wise resting-state functional network centrality alterations in schizophrenia

**DOI:** 10.1038/s41537-023-00422-4

**Published:** 2023-12-16

**Authors:** Lining Guo, Juanwei Ma, Mengjing Cai, Minghui Zhang, Qiang Xu, He Wang, Yijing Zhang, Jia Yao, Zuhao Sun, Yayuan Chen, Hui Xue, Yujie Zhang, Shaoying Wang, Kaizhong Xue, Dan Zhu, Feng Liu

**Affiliations:** 1 Department of Radiology and Tianjin Key Laboratory of Functional Imaging, Tianjin Medical University General Hospital, Tianjin, China; 2 Department of Ultrasound, Tianjin Medical University General Hospital Airport Hospital, Tianjin, China; 3 Department of Radiology and Nuclear Medicine, Xuanwu Hospital, Capital Medical University, Beijing, China; 4 Beijing Key Laboratory of Magnetic Resonance Imaging and Brain Informatics, Beijing, China; 5 Department of Radiology, Tianjin Medical University General Hospital Airport Hospital, Tianjin, China

**Keywords:** Neuroscience, Schizophrenia

## Abstract

Neuroimaging studies have revealed that patients with schizophrenia exhibit disrupted resting-state functional connectivity. However, the inconsistent findings across these studies have hindered our comprehensive understanding of the functional connectivity changes associated with schizophrenia, and the molecular mechanisms associated with these alterations remain largely unclear. A quantitative meta-analysis was first conducted on 21 datasets, involving 1057 patients and 1186 healthy controls, to examine disrupted resting-state functional connectivity in schizophrenia, as measured by whole-brain voxel-wise functional network centrality (FNC). Subsequently, partial least squares regression analysis was employed to investigate the relationship between FNC changes and gene expression profiles obtained from the Allen Human Brain Atlas database. Finally, gene enrichment analysis was performed to unveil the biological significance of the altered FNC-related genes. Compared with healthy controls, patients with schizophrenia show consistently increased FNC in the right inferior parietal cortex extending to the supramarginal gyrus, angular gyrus, bilateral medial prefrontal cortex, and right dorsolateral prefrontal cortex, while decreased FNC in the bilateral insula, bilateral postcentral gyrus, and right inferior temporal gyrus. Meta-regression analysis revealed that increased FNC in the right inferior parietal cortex was positively correlated with clinical score. In addition, these observed functional connectivity changes were found to be spatially associated with the brain-wide expression of specific genes, which were enriched in diverse biological pathways and cell types. These findings highlight the aberrant functional connectivity observed in schizophrenia and its potential molecular underpinnings, providing valuable insights into the neuropathology of dysconnectivity associated with this disorder.

## Introduction

Schizophrenia is a severe mental disorder that poses significant hazards to affected individuals and society. Globally, it is estimated to have a prevalence of approximately 1%, impacting around 26 million people around the world^1^. Schizophrenia is characterized by cognitive and behavioral deficits, including impairments in memory, attention, and emotional processing^2^. Although substantial efforts have been dedicated over the past few decades, the pathophysiological mechanisms of schizophrenia remain elusive due to the inherent heterogeneity in its clinical presentation, varied treatment outcomes, and the complexity of identifying reliable biological markers^3,4^.

Schizophrenia is characterized by dysconnectivity, involving disrupted communication and coordination between different brain regions, which is believed to underlie the cognitive, emotional, and behavioral impairments observed in affected individuals^5^. Resting-state functional magnetic resonance imaging (rs-fMRI) has emerged as a promising tool for investigating the intrinsic functional organization of the brain without the need for specific tasks or stimuli, and various studies have revealed altered resting-state functional connectivity in individuals with schizophrenia^6,7^. Functional connectivity is generally investigated through two approaches, namely seed-based correlation analysis and independent component analysis^8^. However, none of these approaches is able to fully delineate the functional connectivity of the brain^9^. To gain a comprehensive understanding of the impaired resting-state functional connectivity in schizophrenia, an increasing number of neuroimaging studies have adopted the whole-brain voxel-based functional network centrality (FNC) method^10–12^, enabling researchers to explore whole-brain intrinsic connectivity patterns. Over the past few years, the literature has documented resting-state FNC differences in schizophrenia; however, the findings are inconsistent or even contradictory^11,13–16^. The inconsistency among studies may be attributed to differences in sample size, clinical characteristics, approaches to neuroimaging data acquisition, and statistical analyses. Neuroimaging meta-analysis methods have evolved to include a well-defined workflow and a variety of analysis tools to support the integration and analysis of results from neuroimaging studies^17–19^. These tools and workflows enable researchers to integrate neuroimaging data from different studies to gain a comprehensive understanding of neural mechanisms associated with specific brain regions, cognitive functions, or disease states. Despite the potential of neuroimaging meta-analysis to integrate findings from various studies and overcome individual study limitations^20^, there have been no meta-analyses specifically focusing on resting-state FNC in schizophrenia.

Schizophrenia has been recognized as a substantially heritable mental disorder, with an estimated heritability of approximately 80%^21,22^, motivating researchers to conduct genome-wide association studies (GWAS) in search of genetic risk loci associated with this disorder^23,24^. Moreover, there is increasing evidence that resting-state functional connectivity is influenced by genetic factors^25,26^. Despite these findings, the genetic underpinnings underlying functional connectivity changes in schizophrenia remain unclear. It is worth noting that the genetic mechanisms behind the neuroimaging changes in patients with neuropsychiatric disorders cannot be characterized through GWAS, as this approach requires individual phenotypic data. The application of transcription-neuroimaging association analyses has the potential to offer innovative insights into how specific genes may contribute to structural or functional alterations^27–31^. This approach allows us to bridge the gap between macro-level brain connectivity and the micro-level genetic underpinnings of schizophrenia. Leveraging densely sampled gene expression data from the Allen Human Brain Atlas (AHBA), several studies have successfully performed transcriptome-neuroimaging association analyses, uncovering gene expression profiles associated with brain structural and functional changes in patients with schizophrenia^32–35^. However, no studies have been conducted to identify the associations between brain gene expression and resting-state FNC alterations in schizophrenia.

In the present study, neuroimaging and gene expression data were integrated to explore the potential neurobiological genetic mechanisms underlying resting-state FNC differences relevant to schizophrenia. Specifically, a comprehensive coordinate-based meta-analysis was carried out anisotropic effect size version of seed-based *d* mapping (AES-SDM)^36,37^ to identify the robust and consistent whole-brain voxel-wise resting-state FNC changes in schizophrenia. The AES-SDM is a suitable software for neuroimaging meta-analysis, offering flexibility, comprehensiveness, and user-friendliness, while ensuring robust statistical assessments. Then, transcriptome-neuroimaging association analysis was performed to identify genes associated with FNC alterations. Finally, functional annotation and cell type-specific expression analyses (CSEA) were conducted to elucidate the potential biological significance of the identified genes.

## Materials and methods

### Literature search and selection

Two experienced researchers independently conducted a comprehensive and systematic search of relevant studies using PubMed, Web of Science, and Google Scholar databases before May 2023. The search employed the following key terms: [(“schizophrenia” OR “schizoaffective disorder” OR “schizophrenic disorder” OR “disorder, schizophrenic”) AND (“functional connectivity density” OR “FCD” OR “functional connectivity strength” OR“ FCS” OR “degree centrality” OR “DC” OR “voxel-wise network centrality” OR “voxel-wise hub” OR “voxel-wise global brain connectivity” OR “voxel-wise global brain correlation analysis” OR “eigenvector centrality mapping” OR “functional network centrality”) AND (“MRI” OR “neuroimaging” OR “magnetic resonance imaging”) AND (“resting state” OR resting-state” OR “rest”)]. To identify any additional studies, we also manually searched the reference list of the included articles. The studies were included if they met all the following criteria: (1) studies that included patients diagnosed with schizophrenia and a healthy control group; (2) original papers published in peer-reviewed English journals; (3) rs-fMRI studies that explored whole-brain voxel-wise FNC differences in patients with schizophrenia compared to healthy controls; (4) three-dimensional peak coordinates in Montreal Neurological Institute (MNI) or Talairach space were provided; (5) studies with the largest sample sizes were prioritized if the data were obtained from the same resources. Our meta-analysis was conducted following the Preferred Reporting Items for Systematic Reviews and Meta-Analysis (PRISMA) Guidelines^38^.

The quality of each included study was assessed using an 11-point checklist that was adapted from previous meta-analyses^6,39^, with modifications made to reflect critical variables in assessing fMRI studies. The checklist was divided into three categories: subjects (items 1–4), methods for image acquisition and analysis (items 5–9), as well as results and conclusions (items 10 and 11). Each item received a score of 1, 0.5, or 0 when the criteria were fully, partially, or not met, respectively. Of note, the aim of using this checklist was to evaluate the completeness of published studies, not to criticize the investigators or their work. A detailed description of the checklist and the scores assigned to the included studies can be found in Supplementary Tables S1 and S2.

For each study included in the meta-analysis, we recorded relevant variables such as demographic characteristics (i.e., sample size, mean age, and gender), clinical features (i.e., illness duration, medication status, and levels of psychiatric symptom), methodological details (i.e., fMRI data preprocessing and FNC calculation), and statistically significant coordinates and statistics (e.g., *t*-values or other equivalents).

### Voxel-wise meta-analysis

A coordinate-based meta-analysis of FNC changes in schizophrenia was conducted using the AES-SDM software^36,37^ (version 5.15, https://www.sdmproject.com/software). The details of SDM method have been described in detail elsewhere^40^. Briefly, the peak coordinates and *t*-statistics of clusters that showed significant differences in FNC between schizophrenia patients and healthy controls were extracted from each dataset (coordinates reported in Talairach space were converted to MNI space (https://www.sdmproject.com/utilities/?show=Coordinates) and *Z*-values or *P* values were converted to *t*-statistics (https://www.sdmproject.com/utilities/?show=Statistics) by the SDM online converter). If reported statistics were absent, we used a “*p*” to indicate positive (i.e., schizophrenia > controls) peaks and an “*n*” for negative (i.e., schizophrenia < controls) peaks. Subsequently, the extracted peak information was combined to recreate effect size and variance maps for each study. A Gaussian kernel was used to assign higher effect sizes to voxels closer to the peaks, and the full-width at half-maximum (FWHM) was set at 20 mm. Finally, a random-effects meta-analytic model was applied to account for sample size, intra-study variability, and inter-study heterogeneity to generate the mean map by combining individual maps. For an optimal balance false positives and negatives, the statistical significance level was set at voxel-level *P* < 0.005, peak height *Z* > 1, and cluster size > 10^41,42^.

To validate our results, we performed the following experiments: (1) we examined the statistical heterogeneity of significant clusters found in meta-analysis using Cochran’s *Q* statistic. In addition, we utilized funnel plots and conducted Egger’s test to investigate the potential publication bias associated with the significant results. A visually asymmetric funnel plot and *P* < 0.05 in Egger’s test indicate the existence of publication bias in a specific region; (2) a leave-one-out jackknife sensitivity analysis was conducted to assess the reproducibility and robustness of our findings, which consisted of repeating the same meta-analysis after removing each dataset separately. A brain region was deemed robust if it remained significant in most or all combinations of datasets. Currently, there are no established criteria for selecting a threshold in jackknife sensitivity analysis to assess the robustness of the main results^43–45^, and we chose a threshold of 80%; (3) subgroup meta-analyses were undertaken when an adequate number of datasets (>10 datasets) were available to address potential heterogeneity arising from confounding factors. These factors encompassed MRI data acquisition parameters (e.g., time of repetition (TR)/time of echo (TE)), fMRI data preprocessing (including FWHM, and whether the global signal was removed or not), different methods of calculating FNC (binary or weighted), medication status (drug naive/free or not), and MRI scanner type (Philips, General Electric (GE), or Siemens); (4) meta-regression analyses were performed to examine any potential effects of relevant demographic (i.e., mean age of patients and percentage of male patients in the patient group), clinical (i.e., Positive and Negative Syndrome Scale (PANSS) scores (total, positive, or negative score)), and methodological variables (i.e., smoothing kernel, illness duration, and threshold of correlation coefficient of voxel pairs) on between-group FNC differences. To minimize the risk of detecting spurious associations, a more stringent statistical significance level (*P* < 0.0005) was adopted for the meta-regression analyses. Notably, to reduce Type I errors, enhance interpretability, and in line with the research objectives and hypotheses of the study, regions that were not significant in the main analyses were excluded from these meta-regression analyses^37^.

### Transcription-neuroimaging association analysis

Gene expression data were acquired from the AHBA database, consisting of normalized microarray expression data of 20,737 genes from six postmortem brains, with a total of 3702 samples^46^. The demographic information of each donor is available in Supplementary Table S3. The *abagen* toolbox (https://www.github.com/netneurolab/abagen) was employed to extract and process the gene expression data, using the default settings except for the “norm_structures = True” option, which enabled separate normalization for each structural designation (i.e., cortex, subcortex/brainstem, cerebellum)^47^. As a result, a sample-level gene expression matrix (1601 samples × 15,633 genes) was obtained for further analyses.

Partial least squares (PLS) regression was used to identify the pattern of gene expression associated with FNC changes in schizophrenia^48,49^. Thus, to derive the FNC changes of each tissue sample, a 4-mm radius sphere centered at the MNI coordinate of this sample was generated and mean SDM-*Z* value (i.e., FNC change) within the sphere was extracted. In the PLS regression model, each column of the *Z*-score normalized gene expression matrix (1601 samples × 15,633 genes) was taken as the independent variable, and the *Z*-score normalized FNC case–control *t* vector (1601 samples × 1) was treated as the dependent variable. The PLS components, which are a linear combination of weighted gene expression values, are ranked based on the variance explained between the independent and dependent variables. To examine the significance of components from the PLS regression while considering the spatial autocorrelation (SA), we applied Brain Surrogate Maps with Autocorrelated Spatial Heterogeneity approach (BrainSMASH, https://github.com/murraylab/brainsmash)^50^ to generate 1000 surrogate maps preserving the SA of the case–control *Z* vector. Subsequently, all these 1000 surrogate maps were used to generate an empirical null distribution of explained variance of the corresponding PLS component. The *P* value was the proportion of explained variance generated by the surrogate maps that exceeded the explained variance produced by the real data, and the PLS components with *P* < 0.05 were retained. Furthermore, a bootstrapping analysis (1000 times) was conducted to estimate the weight of each gene on the selected component^34^. The normalized weight (*Z*-score), obtained as the ratio of the weight to its standard error, was used to rank the genes based on their contributions to the PLS component. Only the set of genes (positive/negative *Z*-score: PLS+/PLS-) passing Bonferroni correction with *P* < 0.05 (i.e., uncorrected *P* < 0.05/15,633) were used for further enrichment analyses.

To gain insights into the possible biological functions of the significant PLS+/PLS– gene set, we performed gene-category enrichment analysis (GCEA) using the spatial brain phenotype (SBP)-spatial ensemble null models^51^. Specifically, the Gene Ontology (GO) term hierarchy files (data version April 17, 2019) and the corresponding annotation files for Homo sapiens (goa_human.gaf) (https://github.com/benfulcher/GeneSetEnrichmentAnalysis) were downloaded from the Gene Ontology Resource on April 17, 2019 (https://zenodo.org/records/4460714). The analyses were conducted separately using the significant gene sets (PLS+/PLS–) with the following procedures: (1) the category score as the mean loading of the genes in the category was computed, where the loading of each gene was measured as the correlation between the PLS score and the gene expression profile; (2) the null model was constructed by permuting the values of the FNC case–control *Z* vector while preserving SA (1000 permutations); (3) PLS analysis was conducted using the original gene expression matrix and each permuted *Z* vector, and the null gene loadings and null category scores were re-computed; (4) the significance of each term was evaluated by calculating the proportion that permuted category score was higher than the real score. In this analysis, only GO categories with a *P* value below 0.05 were reported.

To determine the specific expression of PLS+/PLS– gene set in different brain cell types, we utilized the online CSEA tool (http://genetics.wustl.edu/jdlab/csea-tool-2/). A specificity index probability (pSI = 0.05) was employed to indicate how genes were more enriched in specific cell type relative to others. For specific expression, Fisher’s exact tests were conducted to evaluate their statistical significance, and multiple testing was corrected using the Benjamini and Hochberg method for false discovery rate (BH-FDR correction) with a corrected *P* value of 0.05.

## Results

### Included studies and sample characteristics

**Fig. 1:**
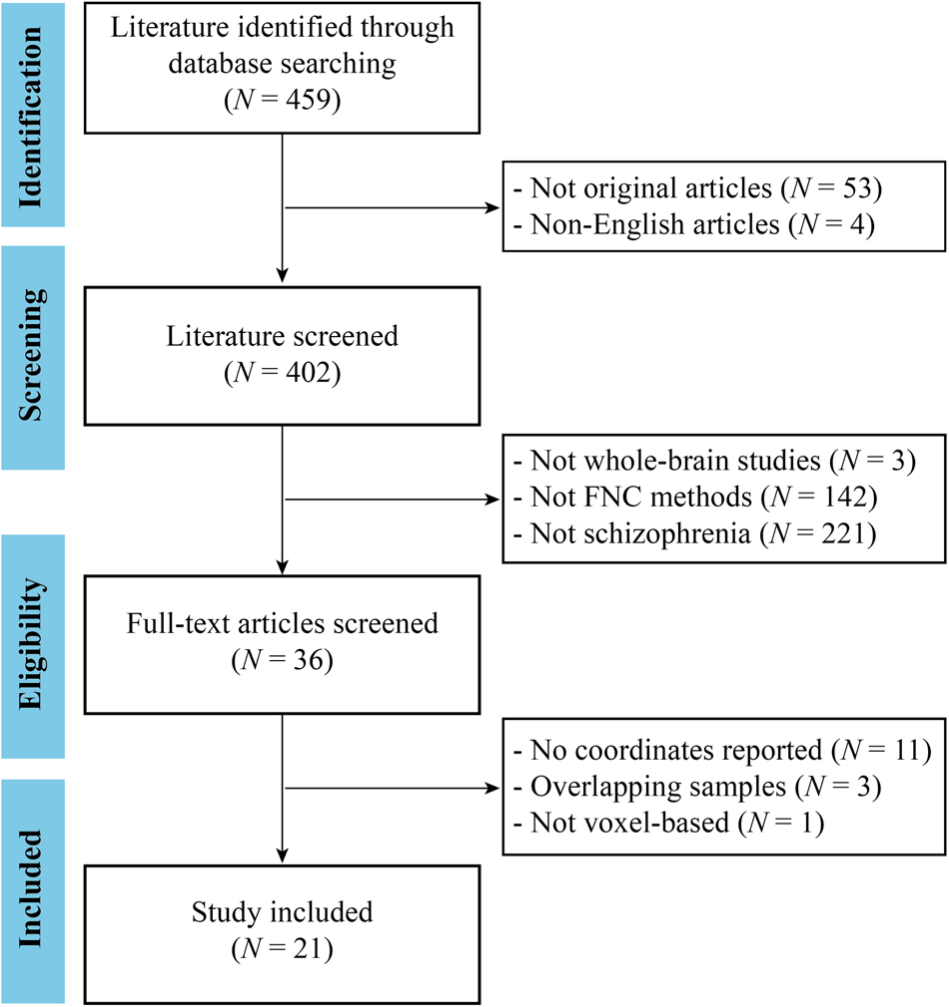
The flowchart of literature search and selection in the meta-analysis. FNC functional network centrality, *N* number.

Prior to conducting this meta-analysis, we assessed all included studies, ensuring that participant selection, data acquisition, preprocessing, and statistical analysis procedures were carried out with rigor in each eligible study. The search strategy identified 459 studies, and 21 of them met our inclusion criteria^10–16,52–65^, comprising a total of 1057 patients with schizophrenia (mean age: 26.1) and 1186 healthy controls (mean age: 27.5). Four of the included studies divided patients with schizophrenia into two subgroups according to clinical characteristics^52,53,55,61^, and thus the two sets of data were obtained from each study. The diagnostic criteria employed in the literature included in our study were predominantly based on widely recognized and accepted classifications, including the Diagnostic and Statistical Manual of Mental Disorders (DSM) and the International Classification of Diseases (ICD). The flow diagram of the identification and screening of studies is shown in Fig. 1, and the clinical and demographic data from all included studies are summarized in Table 1. The mean quality score of included studies was 10.6 (range 10–11), indicating that the included studies were of high quality. A summary of the neuroimaging methodological parameters is shown in Table 2.

**Table 1:** Demographic and clinical characteristics of the studies included in meta-analysis.

Study	Sample size		Mean age (year)		Criteria	Medication status (drug naive/free or not)	Illness duration (month)	PANSS (T/P/N)
	Patients (male)	Controls (male)	Patients	Controls				
Chen et al.^52^ (2015)^a^	42 (19)	84 (42)	24.6	24.7	DSM‑IV	Yes	39.2	86.0/23.2/20.5
Chen et al.^52^ (2015)^a^	42 (21)	84 (42)	24.7	24.7	DSM‑IV	Yes	49.0	86.0/22.7/20.6
Chen et al.^53^ (2022)^b^	46 (21)	48 (23)	21.7	21.9	DSM-V	No	NA	82.3/20.1/20.2
Chen et al.^53^ (2022)^b^	39 (18)	48 (23)	20.2	21.9	DSM-V	No	NA	81.6/18.1/21.0
Chen et al.^54^ (2018)	52 (45)	128 (99)	28.0	27.1	DSM-IV	Yes	71.1	66.5/18.4/17.2
Chen et al.^11^ (2019)	30 (22)	30 (17)	25.8	25	DSM-IV	Yes	51.5	88.0/23.8/22.1
Chen et al.^55^ (2019)^c^	26 (12)	33 (14)	25.1	27.3	SCID-IV	Yes	52.0	44.2/8.8/10.7
Chen et al.^55^ (2019)^c^	31 (14)	33 (14)	24.9	27.3	SCID-IV	Yes	55.6	48.1/13.2/10.9
Ding et al.^56^ (2019)	44 (28)	44 (23)	23.5	23.6	DSM-IV	No	22.3	90.7/22.5/22.5
Guo et al.^57^ (2015)	49 (30)	50 (23)	22.7	23.5	DSM-IV	No	22.5	91.3/22.3/22.8
Guo et al.^58^ (2017)	17 (8)	24 (11)	33.1	30.7	DSM-IV	No	12.0	88.1/23.0/20.2
Kang et al.^59^ (2020)	52 (20)	51 (22)	26.0	26	DSM-IV	No	NA	NA
Li et al.^16^ (2020)	32 (16)	32 (21)	30.9	31.4	DSM-IV	No	8.9	77.4/20.0/20.6
Lei et al.^60^ (2015)	124 (61)	102 (50)	24.5	24.8	DSM-IV	No	6.8	88.4/15.3/15.7
Miao et al.^61^ (2020)^d^	15 (10)	20 (6)	35.2	35.1	DSM-IV	Yes	178.8	71.5/19.3/16.8
Miao et al.^61^ (2020)^d^	19 (7)	20 (6)	34.6	35.1	DSM-IV	Yes	156.0	75.1/20.7/19.8
Palaniyappan et al.^15^ (2014)	39 (30)	34 (23)	34.2	33.8	DSM-IV	Yes	NA	NA
Skåtun et al.^12^ (2016)	71 (44)	196 (113)	28.2	31.5	DSM-IV	Yes	63.6	58.1/13.3/14.8
Wang et al.^62^ (2018)	56 (23)	56 (25)	25.5	25.8	DSM-IV	No	NA	NA
Wang et al.^63^ (2017)	35 (20)	30 (13)	15.5	15.3	DSM-IV-TR	No	16	74.6/20.4/20.9
Yang et al.^64^ (2020)	11 (6)	19 (10)	41.2	37.6	DSM-IV	Yes	NA	84.9/23.6/22.2
Yu et al.^14^ (2021)	22 (11)	60 (38)	33.4	32.9	DSM-IV-TR	No	15.5	93.5/27.2/19.8
Zhao et al.^65^ (2022)	48 (21)	31 (14)	15.8	15.4	DSM-IV-TR	No	5.4	75.1/21.5/17.9
Zhou et al.^10^ (2022)	20 (7)	21 (8)	16.8	16.8	DSM-IV	No	6.0	92.8/24.5/25.7
Zhuo et al.^13^ (2017)	95 (54)	93 (45)	33.6	33	DSM-IV	Yes	121.4	71.5/17.1/20.3

*DSM‑IV* Diagnostic and Statistical Manual of Mental Disorders, Fourth Edition, *DSM-V* Diagnostic and Statistical Manual of Mental Disorders, Fifth Edition, *DSM-IV-TR* Diagnostic and Statistical Manual of Mental Disorders, Fourth Edition, Text Revision, *N* negative symptom score, *NA* not available, *P* positive symptom score, *PANSS* Positive and Negative Syndrome Scale, *SCID-IV* Structured Clinical Interview for the Diagnostic and Statistical Manual of Mental Disorders, Fourth Edition, *T* total symptom score.

^a–d^The four studies divided patients into two datasets based on different symptoms.

**Table 2:** Technique details of the studies included in the meta-analysis.

Study	Scanner	Sequence	GS removed	FWHM (mm)	TR (ms)	TE (ms)	ST (mm)	Correlation coefficient	Binary/weighted FNC	Threshold	Number of coordinates
Chen et al.^52^ (2015)^a^	GE	EPI	Yes	6	2000	30	4	0.25	Weighted	*P* < 0.01 (corrected)	3
Chen et al.^52^ (2015)^a^	GE	EPI	Yes	6	2000	30	4	0.25	Weighted	*P* < 0.01 (corrected)	3
Chen et al.^53^ (2022)^b^	GE	GRE-SS-EPI	No	6	2000	30	4	0.2	Weighted	*P* < 0.05 (corrected)	4
Chen et al.^53^ (2022)^b^	GE	GRE-SS-EPI	No	6	2000	30	4	0.2	Weighted	*P* < 0.05 (corrected)	7
Chen et al.^54^ (2018)	Siemens	EPI	No	4	2000	30	4	0.2	Binary	*P* < 0.05 (corrected)	1
Chen et al.^11^ (2019)	Siemens	EPI	No	6	2000	30	4	0.2	Binary	*P* < 0.05 (corrected)	6
Chen et al.^55^ (2019)^c^	GE	Gradient-recalled EPI	Yes	4	2000	35	3.6	0.25	Weighted	*P* < 0.05 (corrected)	7
Chen et al.^55^ (2019)^c^	GE	Gradient-recalled EPI	Yes	4	2000	35	3.6	0.25	Weighted	*P* < 0.05 (corrected)	4
Ding et al.^56^ (2019)	Siemens	GRE-EPI	No	4	2000	30	4	NA	Weighted	*P* < 0.05 (corrected)	7
Guo et al.^57^ (2015)	Siemens	GRE-EPI	No	8	2000	30	4	NA	Weighted	*P* < 0.001 (corrected)	3
Guo et al.^58^ (2017)	Philips	GRE-EPI	No	4	2000	30	5	NA	Weighted	*P* < 0.001 (corrected)	6
Kang et al.^59^ (2020)	Siemens	EPI	No	6	3000	30	3	0.6	Binary	*P* < 0.05 (corrected)	5
Li et al.^16^ (2020)	Siemens	GRE-EPI	No	NA	2000	30	4	NA	Weighted	*P* < 0.05 (corrected)	4
Lei et al.^60^ (2015)	GE	EPI	Yes	6	2000	30	5	0.25	Binary	*P* < 0.05 (corrected)	2
Miao et al.^61^ (2020)^d^	GE	NA	No	4	2000	40	5	0.2	Weighted	*P* < 0.05 (corrected)	7
Miao et al.^61^ (2020)^d^	GE	NA	No	4	2000	40	5	0.2	Weighted	*P* < 0.05 (corrected)	4
Palaniyappan et al.^15^ (2014)	Philips	GRE-EPI	Yes	8	2500	25/53	4	0.25	Binary	*P* < 0.05 (corrected)	13
Skåtun et al.^12^ (2016)	GE	GRE-EPI	No	6	2638	30	3	NA	Binary	*P* < 0.05 (corrected)	12
Wang et al.^62^ (2018)	Siemens	EPI	No	8	3000	30	3	0.6	Binary	*P* < 0.05 (corrected)	2
Wang et al.^63^ (2017)	Siemens	EPI	No	6	2000	30	4	FWE correction	Binary	*P* < 0.05 (corrected)	7
Yang et al.^64^ (2020)	Phillips	EPI	No	6	2000	30	4	0.3	Binary	*P* < 0.005 (uncorrected)	2
Yu et al.^14^ (2021)	Siemens	GRE-EPI	No	6	2000	30	4	0.25	Binary	*P* < 0.05 (corrected)	4
Zhao et al.^65^ (2022)	Siemens	EPI	No	4	2000	30	NA	NA	Weighted	*P* < 0.05 (corrected)	5
Zhou et al.^10^ (2022)	Siemens	GRE-EPI	No	8	2000	30	4	0.25	Weighted	*P* < 0.05 (corrected)	3
Zhuo et al.^13^ (2017)	GE	GRE-SS-EPI	No	6	2000	45	4	0.6	Binary	*P* < 0.05 (corrected)	13

*EPI* echo planar imaging, *FNC* functional network centrality, *FWE* family-wise error, *FWHM* full-width at half-maximum, *GE* General Electric, *GRE* gradient echo, *GS* global signal, *NA* not available, *SS* single shot, *ST* slice thickness, *TE* time of echo, *TR* time of repetition.

^a–d^The four studies divided patients into two datasets based on different symptoms.

### FNC changes in meta-analysis

**Fig. 2:**
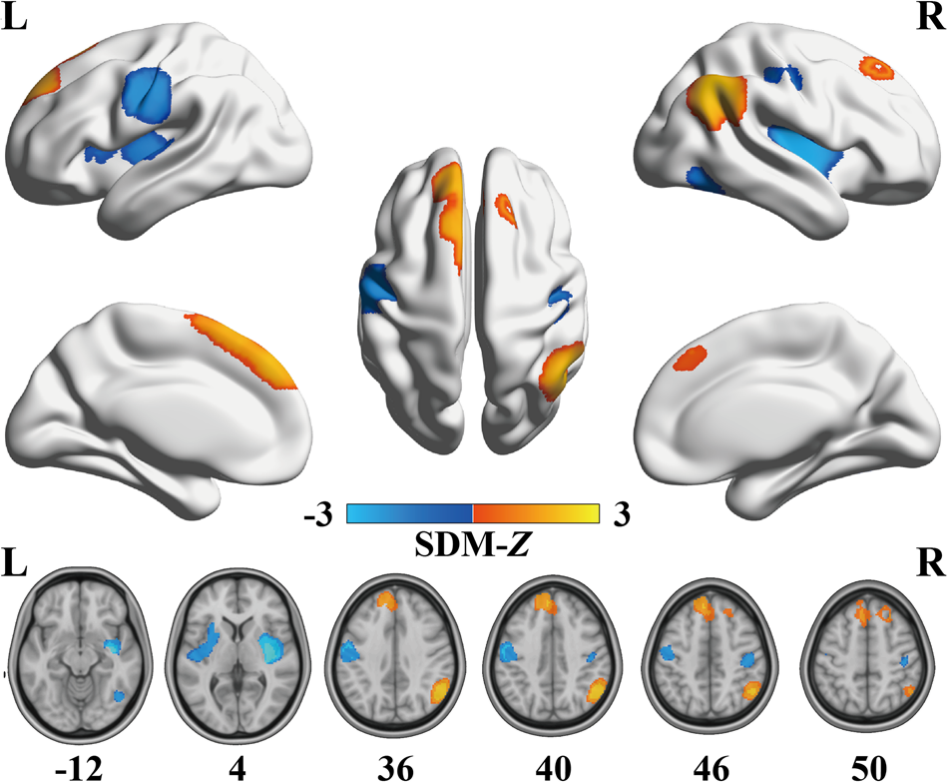
Regions of significantly increased (warm color) and decreased (cold color) functional network centrality in schizophrenia patients in the pooled meta-analysis. L left, R right, SDM seed-based *d* mapping.

In schizophrenia patients, significant increases in FNC were observed in the right inferior parietal cortex (IPC) extending to the supramarginal gyrus (SMG) and angular gyrus (ANG), bilateral medial prefrontal cortex (mPFC), and right dorsolateral prefrontal cortex (DLPFC). In addition, significant decreases in FNC were found in the bilateral insula, bilateral postcentral gyrus (PCG), and right inferior temporal gyrus (ITG) (Table 3 and Fig. 2).

**Table 3:** FNC changes in patients with schizophrenia in the meta-analysis.

Regions	Peak MNI coordinates			Cluster size (voxels)	SDM-*Z* value	*P* value	Heterogeneity test	Egger’s test (*P* value)
	*x*	*y*	*z*				*Q* (*P* value)	
Increased FNCs								
Right IPC/SMG/ANG	54	–54	36	1497	2.297	<0.0001	23.193 (0.508)	0.222
Bilateral mPFC	–6	42	44	1620	2.118	<0.0001	18.494 (0.778)	0.870
Right DLPFC	24	26	50	183	1.579	0.0007	20.714 (0.656)	0.095
Decreased FNCs								
Right insula	38	–8	–10	2354	–3.052	<0.0001	23.920 (0.466)	0.460
Left insula	–30	6	4	1140	–1.767	0.0009	25.588 (0.374)	0.104
Left PCG	–58	–8	40	963	–2.060	0.0002	21.866 (0.587)	0.737
Right PCG	44	–22	46	238	–1.746	0.0010	18.776 (0.764)	0.592
Right ITG	46	–64	–12	206	–1.796	0.0008	19.944 (0.700)	0.302

*ANG* angular gyrus, *DLPFC* dorsolateral prefrontal cortex, *FNC* functional network centrality, *IPC* inferior parietal cortex, *ITG* inferior temporal gyrus, *MNI* Montreal Neurological Institute, *mPFC* medial prefrontal cortex, *PCG* postcentral gyrus, *Q* Cochran’s *Q* statistic, *SDM* seed-based *d* mapping, *SMG* supramarginal gyrus.

**Fig. 3:**
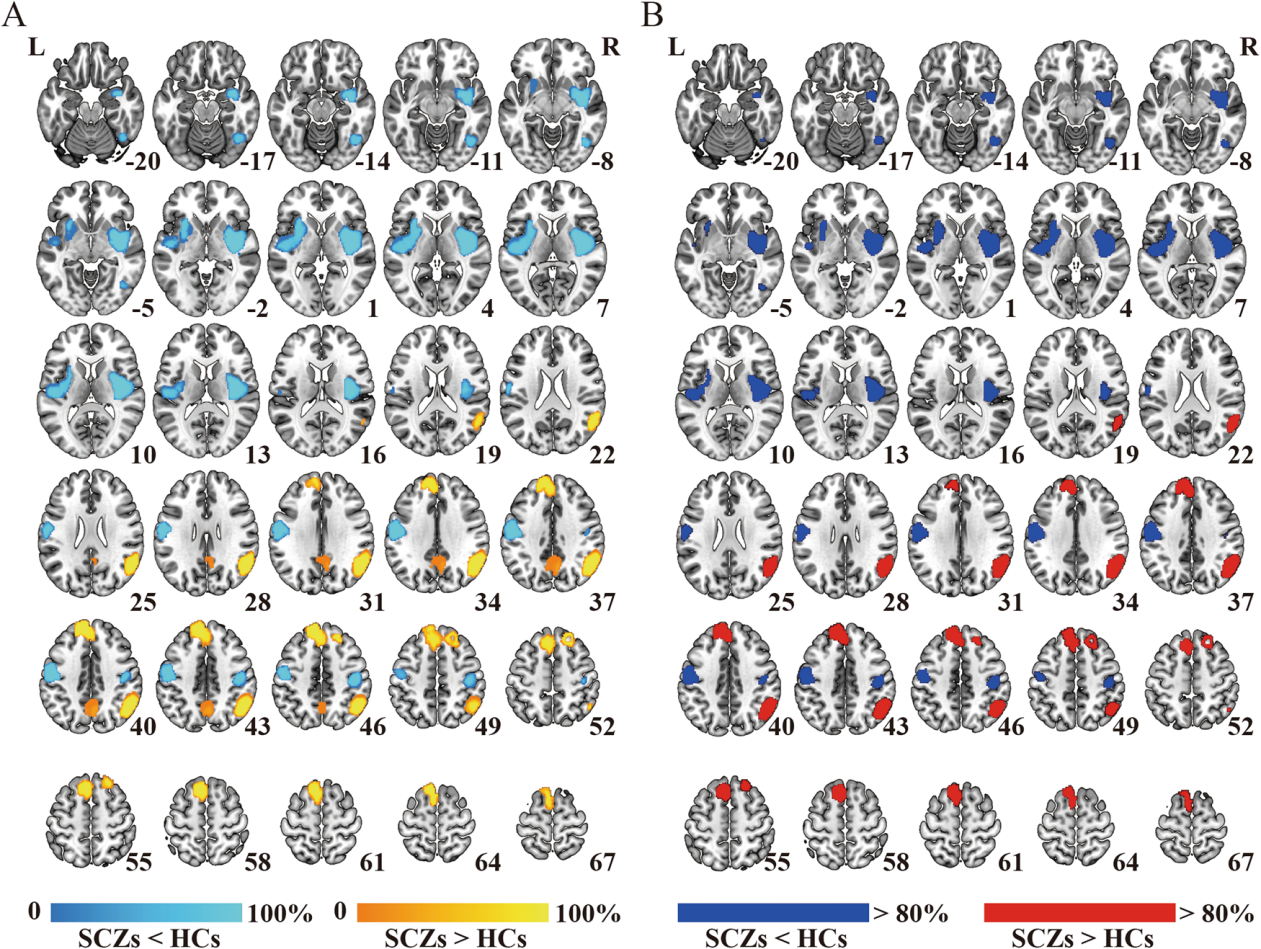
Results of the jackknife sensitivity analysis. **A** The voxel-wise probability map uncovers clusters that are significant in jackknife analysis, and the value for each voxel corresponds to the probability of occurrence in all iterations. **B** Regions that were replicated in more than 80% of the iterations. HC healthy control, L left, R right, SCZ schizophrenia patient.

**Fig. 4:**
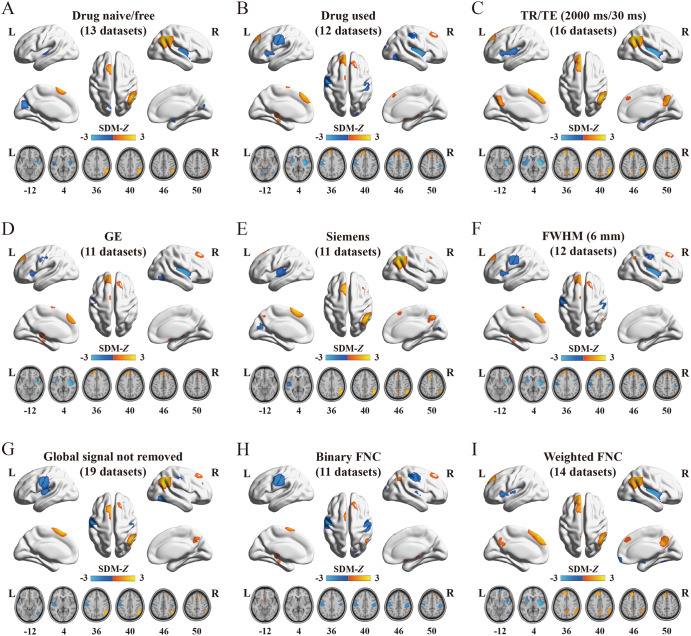
Regions of significantly altered functional network centrality in nine specific subgroups. **A** Studies involving drug naive/free patients, **B** studies involving patients under antipsychotic drug treatment, **C** studies with TR = 2000 ms, TE = 30 ms, **D** studies employing GE MRI scanners, **E** studies utilizing Siemens MRI scanners, **F** studies using a 6 mm FWHM smooth kernel, **G** studies where global signal was not regressed out, **H** studies examining binary FNC and **I** studies analyzing weighted FNC. FNC functional network centrality, FWHM full-width half-maximum, GE General Electric, L left, MRI magnetic resonance imaging, R right, SDM seed-based *d* mapping, TE time of echo, TR time of repetition.

**Fig. 5:**
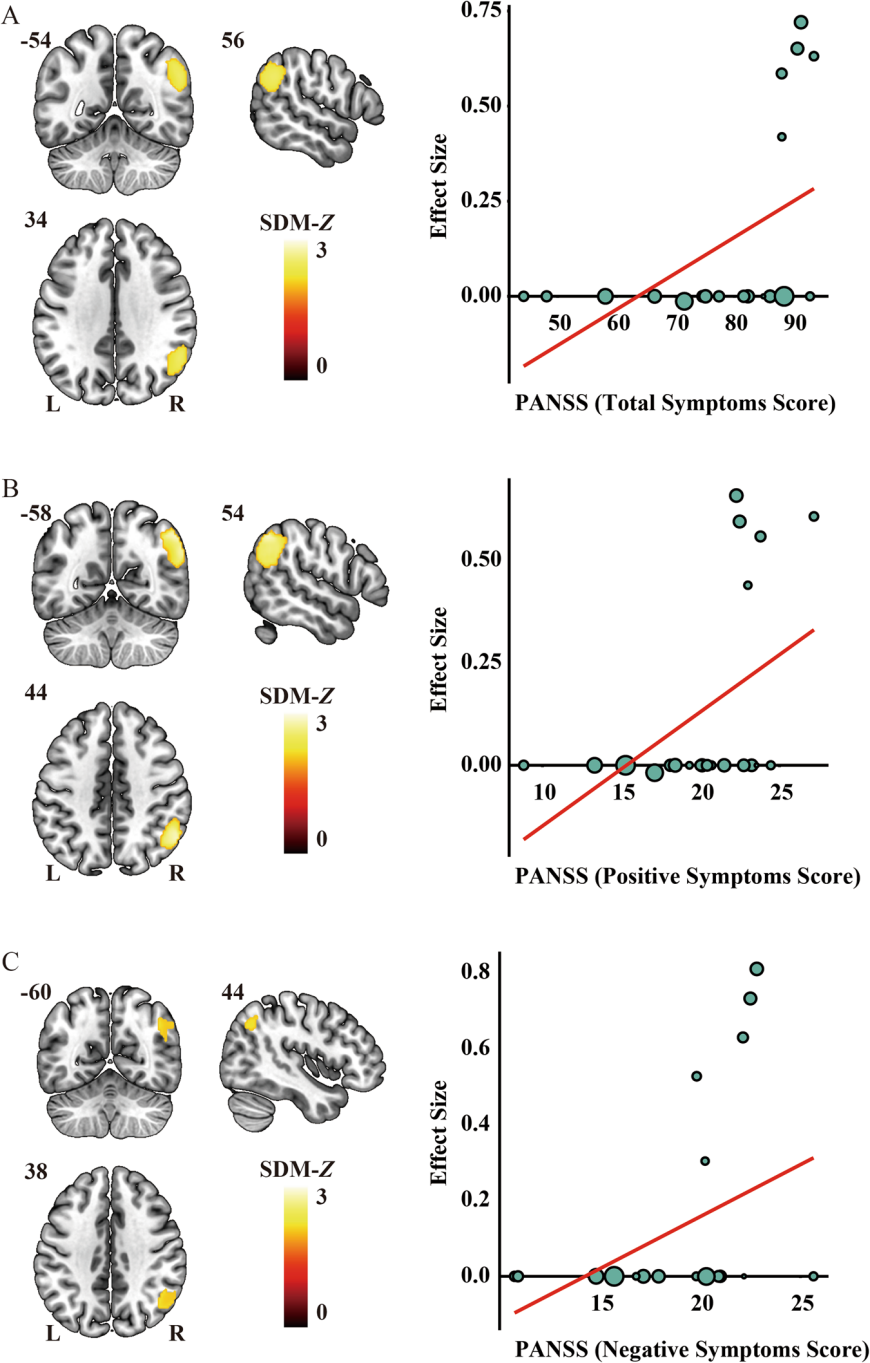
Significant results of meta-regression analyses. PANSS scores for total (**A**, right), positive (**B**, right), and negative (**C**, right) symptom score in schizophrenia patients were positively associated with altered FNC in the right IPC (**A**–**C**, left). In the scatterplot, each data point signifies a dataset, where larger data points correspond to larger sample sizes. L left, PANSS Positive and Negative Syndrome Scale, R right, SDM seed-based *d* mapping.

A set of validation analyses was conducted to confirm the validity of the results. First, the heterogeneity analyses indicated the absence of significant heterogeneity within each significant cluster among the studies. In addition, both Egger’s tests and the funnel plot results demonstrated no signs of publication bias, as shown in Table 3 and Supplementary Fig. S1. Second, jackknife sensitivity analysis demonstrated high replicability and reproducibility of the pooled meta-analysis results, as all clusters were consistently identified in more than 80% of iterations (Supplementary Table S4 and Fig. 3). Third, although slight differences were observed between the results of subgroup analyses and the pooled meta-analysis, the findings of the subgroup analyses remained largely consistent (Supplementary Table S5 and Fig. 4). Finally, in the meta-regression analyses, the results indicated that PANSS scores in schizophrenia patients were positively associated with altered FNC in the right IPC (total symptom score: peak MNI coordinate: *x* = 56, *y* = –54, *z* = 34, 754 voxels, SDM-*Z* = 2.506, *P* < 0.0001; positive symptom score: peak MNI coordinate: *x* = 54, *y* = –58, *z* = 44, 989 voxels, SDM-*Z* = 2.809, *P* < 0.0001; negative symptom score: peak MNI coordinate: *x* = 44, *y* = –60, *z* = 38, 305 voxels, SDM-*Z* = 2.198, *P* < 0.0001) (Fig. 5). We re-performed the meta-regression analysis after removing studies with null effect sizes, and the results also confirmed a significant positive association between altered FNC in the right IPC and PANSS scores in schizophrenia patients (Supplementary Fig. S2). However, the mean age of patients, percentage of male patients, illness duration, smoothing kernel, and threshold of the correlation coefficient of voxel pairs were not found to be correlated with FNC changes, at least linearly.

### Genes associated with FNC alterations

**Fig. 6:**
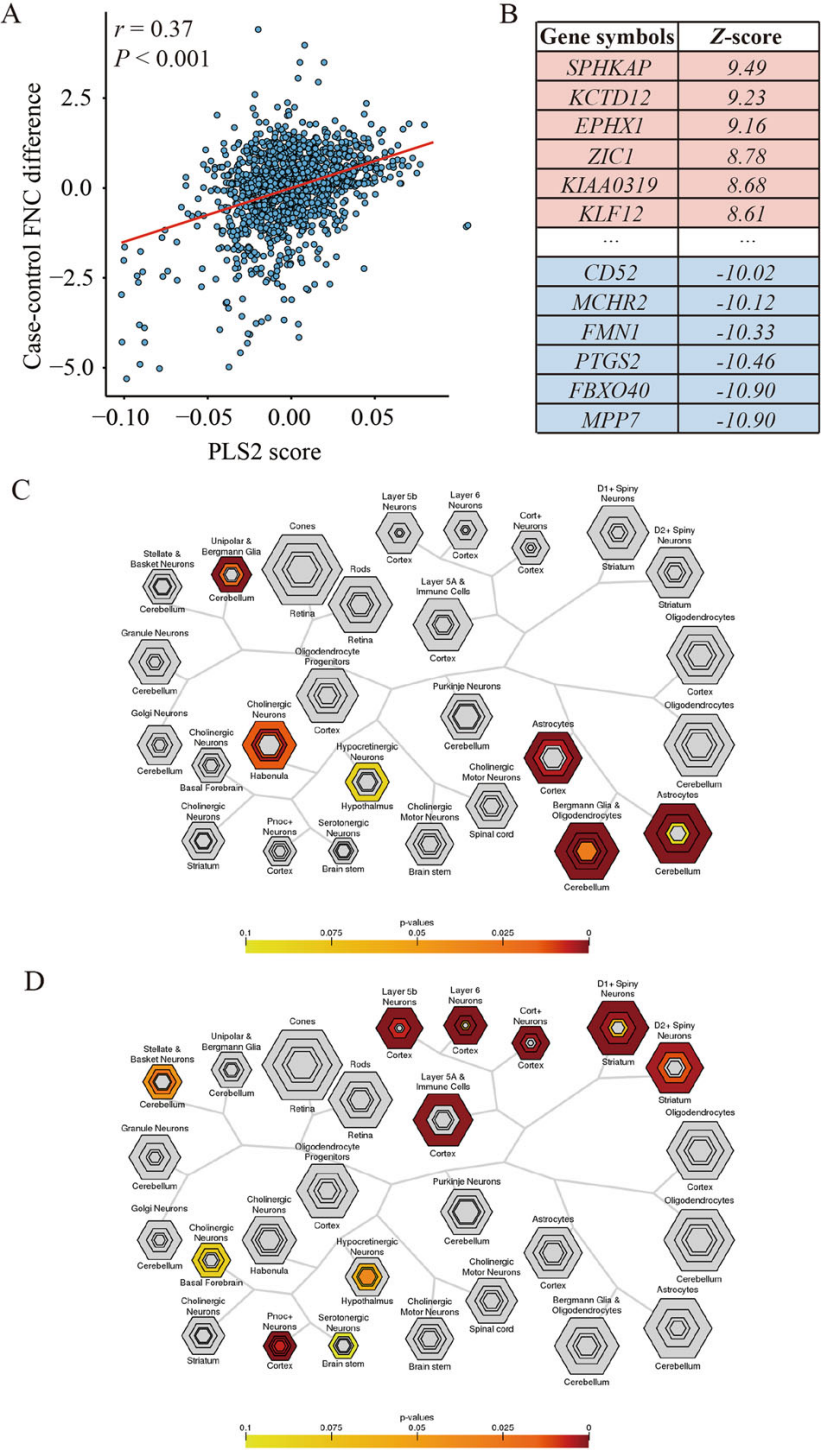
Gene expression profiles related to FNC differences and the results of enrichment analyses. **A** Scatterplot represents the relationship between PLS2 score and case–control FNC differences. Each point denotes a tissue sample. **B** Ranked PLS2 genes based on *Z*-score. **C** Cell type-specific expression analyses using the PLS2+ gene list. **D** Cell type-specific expression analyses using the PLS2- gene list. Colored cell types represent significance after multiple testing correction. The sizes of the hexagons denote cell-type specificity across different specificity index probability (pSI) statistic thresholds ranging from 0.05 to 1e-4. The outer hexagons correspond to the least specific test for a cell type (pSI threshold = 0.05), whereas the innermost hexagon reflects the most specific test for a cell type (pSI threshold = 1e-4). FNC functional network centrality, PLS partial least squares.

As shown in Supplementary Fig. S3, the top five components derived from the PLS regression explained 12.31%, 14.01%, 6.63%, 5.52% and 5.39% of the variance. Among them, only the second PLS component (PLS2) significantly accounted for more variance in the FNC case–control differences than expected by chance (*P* = 0.043). The PLS2 gene expression score was positively correlated with the case–control *Z* vector (Pearson’s *r* = 0.374, *P* < 0.001, permutation test with SA corrected, 1000 permutations, Fig. 6A). After the bootstrapping analysis, we identified 1060 genes that made significant contributions to PLS2 (*P* < 0.05, Bonferroni corrected). Within this set, 485 genes had positive PLS2 weights (PLS2+), while 575 genes had negative PLS2 weights (PLS2–) (Supplementary Table S6 and Fig. 6B).

After correcting for multiple comparisons, no significant enrichment in functional annotations was observed for the PLS2+ genes. In contrast, the PLS2– gene set exhibited preferential enrichment in several processes (Supplementary Table S7), including apoptotic process, response to estradiol, and response to vitamin D. The CSEA results indicated that PLS2+ genes were mainly expressed in cortical cells (e.g., astrocytes) and cerebellar cells (e.g., unipolar and Bergmann glia, Bergmann glia, and oligodendrocytes). On the other hand, PLS2– genes showed specific expression in cortical neurons (e.g., Pnoc+, Ntsr+, Glt25d2, and Cort+) and immune cell (Fig. 6C, D).

## Discussion

This preliminary study investigated whole-brain voxel-wise resting-state FNC differences and their associated genetic architecture in schizophrenia. Meta-analysis revealed robust and replicable FNC pattern of differences in schizophrenia patients. FNC was significantly increased in the bilateral mPFC, right IPC and right DLPFC and significantly reduced in the bilateral insula, bilateral PCG, and right ITG. Meta-regression analysis revealed that increased FNC in the right IPC was positively correlated with PANSS scores. Furthermore, transcription-neuroimaging analysis indicated gene sets that may contribute to molecular mechanisms of schizophrenia by modulating FNC, and these genes involved in distinct functional annotations and cell types. Overall, these findings establish connections that link specific genes, biological processes, and cell types to differences in FNC observed in schizophrenia, thereby providing preliminary evidence of the potential neurobiological mechanisms underlying FNC changes in schizophrenia.

Significantly increased FNC was observed in the bilateral mPFC, right IPC and right DLPFC. The mPFC, a critical cortical region, integrates information from multiple cortical and subcortical area and brings updated information together to form output structures^66^. It is pivotal for various brain functions, such as cognition, emotion regulation, motivation, and sociability. Previous studies have indicated that a reduction in dopamine release in the mPFC is a hallmark of schizophrenia pathophysiology^67^, increased FNC in this region may reflect a compensatory mechanism for maintaining the normal dopamine release in schizophrenia. In addition, the IPC is involved in several neuropsychological functions affected in schizophrenia and supports the frontal lobe in storing and retrieving verbal information^68^. Functional differences of the IPC in schizophrenia have been widely noted^69^. For example, Guo et al. found that the connections involving the IPC were partially strengthened in schizophrenia patients and were positively correlated with PANSS scores^70^. The observation in our study, which reveals disrupted connections in the IPC and their positive correlation with PANSS scores, further supports the significant role of this region in symptom severity. Subgroup analysis revealed FNC changes in the IPC specifically in the drug-used group, indicating that IPC could be a potential target for treatment interventions in schizophrenia. Regarding the DLPFC, extensive research has demonstrated its pivotal role in cognitive function and the physiologic dysfunction in schizophrenia^71,72^, and the results of our study confirmed these earlier findings.

In parallel, we observed decreased FNC values in the bilateral insula, bilateral PCG, and right ITG in schizophrenia patients compared to healthy controls. The insula cortex, a cortical structure with extensive connections to different parts of the cortex and the limbic system, has become a focal point for schizophrenia research^73^. Disruption in insula processing may contribute to many of the sensory deficits observed in schizophrenia^74^. Our results supported that insula functional connectivity was overall reduced in schizophrenia^75^. Moving on to the sensorimotor network, which encompasses somatosensory (postcentral gyrus) and motor (precentral gyrus) regions and extends to the supplementary motor areas, which are responsible for sensory processing and motor functions^76,77^. Our findings are consistent with previous studies that have reported functional dysconnectivity in sensory-motor areas in patients with schizophrenia^78,79^. Sensorimotor abnormalities act as vulnerability markers in individuals with psychosis risk syndrome^80,81^. Sensorimotor signs and symptoms are predictors of the clinical course in patients with first-episode schizophrenia^82^. Considering schizophrenia’s severe nature and its impact on various domains, including perception and motor function, dysfunction within the sensorimotor network could potentially lead to abnormal sensorimotor integration, representing a plausible dysfunctional mechanism underlying schizophrenia. Furthermore, we observed aberrant FNC in the ITG, which is a component of the visual network responsible for visuospatial perception^83^. Importantly, visual processing impairment is a well-recognized abnormality in individuals with schizophrenia^84^. Therefore, our findings highlight the potential involvement of the visual processing regions in the underlying mechanisms of schizophrenia.

Transcription-neuroimaging association analysis identified two gene sets (i.e., PLS2+ and PLS2–) that exhibited correlations with alterations in resting-state FNC in schizophrenia. The GCEA approach further identified genes within the PLS2– set enriched for processes related to apoptosis, responses to estradiol, and responses to vitamin D. During the course of synaptic overpruning, emerging evidence suggests that dendritic apoptosis, along with disrupted synaptic plasticity, contributes to the formation of neuronal misconnections, which are thought to underlie the primary negative symptoms and cognitive deficits observed in schizophrenia^85^. In addition, the varying symptoms and severity of schizophrenia among men and women suggest a potential protective role of estrogen^86^. Increased estradiol levels during menstruation and pregnancy have been associated with reduced mental illness severity. These preliminary findings have already led to the utilization of hormone replacement therapy in clinical settings^87^. Moreover, an epidemiological study has highlighted the link between vitamin D deficiency and schizophrenia^88^. Nevertheless, no significant enrichment in functional annotations was detected for the PLS2+ genes. Therefore, the PLS2– provides a possible mechanistic explanation for the FNC differences observed in schizophrenia via the above biological pathways.

The CSEA results indicated that PLS2+ genes exhibited specific expression in the cerebellum, whereas PLS2– genes demonstrated expression in the cortex. This also confirmed that schizophrenia primarily affects the cerebral cortex, which is influenced by the activity of the cerebellum^89^. Our findings further suggest that PLS2+ genes were predominantly enriched in glial cells, particularly astrocytes, which are known to play a pivotal role in the molecular and functional deviations underlying the pathophysiology of schizophrenia^90^. Astrocytes influence crucial aspects such as glutamatergic signaling, synaptogenesis, synaptic pruning, and myelination. These dysregulated processes contribute to the complex and multifaceted nature of schizophrenia’s etiology^91^. The PLS2– genes showed exclusive expression in neurons, where the dopamine system is presumed to exert a noteworthy influence. Dopamine is a neurotransmitter associated with various aspects of emotion, behavior, and reward system. This excessive dopamine activity might be linked to neurons being overly excited. Furthermore, these genes demonstrated specific expression in immune cells, which aligns with prior research indicating a potential connection between cognitive impairments in psychosis and altered expression of molecules involved in immune cell trafficking^92^. Based on the above enrichment results, it is suggested that the PLS2– may provide a promising genetic resource for investigating the FNC changes in individuals with schizophrenia.

Several limitations are worth mentioning in this study. First, the coordinate-based meta-analysis relies on summarizing reported local peak coordinates instead of raw statistical maps, which can potentially lead to less precise results. Second, despite the AHBA was derived from only six adult donors, there is a much greater variability in gene expression across brain regions than across the six individuals, which indicating that the AHBA can be robustly used for investigation purposes. Nevertheless, concerns about the donor brains’ representativeness remain valid^93^. Third, due to AHBA data relying on postmortem measurements from a limited sample, any associations discovered between gene expression patterns and imaging phenotypes are purely correlational and not causal. Fourth, the absence of direct experimental validation for the identified genetic targets and their functional roles in altering FNC limits the conclusive understanding of their therapeutic potential. Experimental confirmation through animal studies targeting these genetic elements is crucial to validate these preliminary findings and assess their viability as therapeutic targets in schizophrenia. Fifth, head motion in rs-fMRI may adversely affect the quality and interpretability of the acquired data^94^. While the original studies did include quality checks and head motion correction, it’s essential to acknowledge that the potential influence of head motion may not have been entirely eliminated. Finally, the neuroimaging metrics indirectly reflect brain structure and function and can be influenced by numerous potential confounding factors, including hydration, blood lipid levels, and cortisol levels^95^. Therefore, we should approach the interpretation of the results with caution.

## Conclusion

In summary, our meta-analysis revealed the consistent whole-brain voxel-wise resting-state FNC alterations in patients with schizophrenia. In addition, we found positive correlations between higher PANSS scores for total, positive, and negative symptoms and increased FNC in the right IPC. Moreover, the FNC differences pattern in schizophrenia showed spatial correlations with two distinct gene sets enriched for diverse biological pathways and different cell types. These findings preliminarily shed light on the pathophysiological mechanisms underlying schizophrenia, and further suggest a complex polygenic and multi-pathway architecture of this disorder.

### Supplementary information

Supplementary Material

## Data Availability

All result files for the current meta-analysis are publicly available at figshare (10.6084/m9.figshare.24085980.v1) and the results for the transcription-neuroimaging association analysis are provided in the supplementary material.
