# Supplementary material for: Transcriptional signatures of the whole-brain voxel-wise resting-state functional network centrality alterations in schizophrenia
Source: Schizophrenia (Heidelb). 2023 Dec 16;9(1):87. doi: 10.1038/s41537-023-00422-4 (PMC10725456; doi:10.1038/s41537-023-00422-4)
Supplement: Supplementary file 1 — Supplementary Material [file 41537_2023_422_MOESM1_ESM.docx]

**Online supplementary material**

**Transcriptional signatures of the whole-brain voxel-wise resting-state functional network centrality alterations in schizophrenia**

**Lining Guo^1, a^, Juanwei Ma^1, a^, Mengjing Cai^1, a^, Minghui Zhang^2^, Qiang Xu^1^, He Wang^1^, Yijing Zhang^1^, Jia Yao^1^, Zuhao Sun^1^, Yayuan Chen^1^, Hui Xue^1^, Yujie Zhang^1^, Shaoying Wang^1^, Kaizhong Xue^1, 3, 4*^, Dan Zhu^1, 5*^, Feng Liu^1*^**

^1^Department of Radiology and Tianjin Key Laboratory of Functional Imaging, Tianjin Medical University General Hospital, Tianjin, China.

^2^Department of Ultrasound, Tianjin Medical University General Hospital Airport Hospital, Tianjin, China.

^3^Department of Radiology and Nuclear Medicine, Xuanwu Hospital, Capital Medical University, Beijing, China.

^4^Beijing Key Laboratory of Magnetic Resonance Imaging and Brain Informatics, Beijing, China.

^5^Department of Radiology, Tianjin Medical University General Hospital Airport Hospital, Tianjin, China.

^a^ Lining Guo, Juanwei Ma, and Mengjing Cai contributed equally to this work.

^*^ **Correspondence to:**

Feng Liu, PhD

Department of Radiology and Tianjin Key Laboratory of Functional Imaging, Tianjin Medical University General Hospital, Tianjin 300052, China.

E-mail: fengliu@tmu.edu.cn

Dan Zhu, PhD

Department of Radiology and Tianjin Key Laboratory of Functional Imaging, Tianjin Medical University General Hospital, Tianjin 300052, China.

Department of Radiology, Tianjin Medical University General Hospital Airport Hospital, Tianjin, 300308, China.

E-mail: zhudan@tmu.edu.cn

Kaizhong Xue, MD

Department of Radiology and Tianjin Key Laboratory of Functional Imaging, Tianjin Medical University General Hospital, Tianjin 300052, China.

Department of Radiology and Nuclear Medicine, Xuanwu Hospital, Capital Medical University, Beijing, China.

Beijing Key Laboratory of Magnetic Resonance Imaging and Brain Informatics, Beijing, China.

E-mail: xuekaizhong@tmu.edu.cn

**Details of gene expression data preprocessing**

The specific preprocessing steps of gene expression data can be summarized as flollows^1^: (1) probe-to-gene annotations were updated: microarray probes were reannotated with information generated by Arnatkevičiūtė et al.^1^ and those unreliable probes that could not be matched to genes were discarded; (2) intensity-based filter: as suggested by the previously published pipeline, the annotated probes were filtered according to their intensity that did not exceed background noise in 50% of all tissue samples; (3) probe selection: a single microarray probe with the highest differential stability was selected to represent each gene, which retained 15,633 probes representing 15,633 genes; (4) MNI coordinates correction: MNI coordinates of tissue samples from six donors were corrected using the *alleninf* package; (5) expression values normalization: a scaled robust sigmoid function was used to normalize expression values for each sample and donor across genes, and also for each gene and donor across samples; (6) brain structure normalization: normalization was performed within structural classes cortex, subcortical/brain stem and cerebellum to account for differences in gene expression patterns between these brain structures. Thus, the sample-level gene expression matrix (1,601 samples × 15,633 genes) was finally used in our subsequent analyses (more details of six donors in the AHBA database are shown in Table S3).

**References**

1 Arnatkeviciute A, Fulcher BD and Fornito A. A practical guide to linking brain-wide gene expression and neuroimaging data. Neuroimage 2019; 189: 353-367

**Supplementary Tables**

Table S1. The checklist of quality assessment for included studies.

| **Category 1: Subjects**   **Score (0/0.5/1)** |
| --- |
| 1. Patients were evaluated prospectively, specific diagnostic criteria were applied, and demographic data was reported |
| 2. Healthy comparison subjects were evaluated prospectively, and psychiatric and medical illnesses were excluded |
| 3. Important variables (e.g., age, gender, illness duration, medication status, comorbidity, and severity of illness) were checked, either by stratification or statistically |
| 4. Sample size per group > 10 |
| **Category 2: Methods for image acquisition and analysis** |
| 5. Magnetic field strength ≥ 1.5 T |
| 6. Whole brain analysis was automated with no *a* *priori* regional selection |
| 7. Coordinates reported in a standard space |
| 8. The imaging processing technique was described clearly enough to be reproduced |
| 9. Measurements were described clearly enough to be reproduced |
| **Category 3: Results and conclusions** |
| 10. Statistical parameters were presented for both significant and noteworthy non-significant differences. |
| 11. Conclusions were consistent with the results obtained and the limitations were discussed |
| **Total score 11** |

Abbreviations: fMRI, functional magnetic resonance imaging; T, tesla.

Table S2. The quality assessment scores of included studies.

| **Study** | **Item 1** | **Item 2** | **Item 3** | **Item 4** | **Item 5** | **Item 6** | **Item 7** | **Item 8** | **Item 9** | **Item 10** | **Item 11** | **Total** |
| --- | --- | --- | --- | --- | --- | --- | --- | --- | --- | --- | --- | --- |
| Chen C et al. (2015) | 1 | 1 | 1 | 1 | 1 | 1 | 1 | 1 | 1 | 1 | 1 | 11.0 |
| Chen J et al. (2022) | 1 | 1 | 0.5 | 1 | 1 | 1 | 1 | 1 | 1 | 1 | 0.5 | 10.0 |
| Chen X et al. (2018) | 1 | 1 | 1 | 1 | 1 | 1 | 1 | 1 | 1 | 1 | 1 | 11.0 |
| Chen X et al. (2019) | 1 | 1 | 1 | 1 | 1 | 1 | 1 | 1 | 1 | 1 | 0.5 | 10.5 |
| Chen X et al. (2019) | 1 | 1 | 1 | 1 | 1 | 1 | 1 | 1 | 1 | 1 | 1 | 11.0 |
| Ding Y et al. (2019) | 1 | 1 | 1 | 1 | 1 | 1 | 1 | 1 | 0.5 | 1 | 1 | 10.5 |
| Guo W et al. (2015) | 1 | 1 | 1 | 1 | 1 | 1 | 1 | 1 | 0.5 | 1 | 1 | 10.5 |
| Guo W et al. (2017) | 1 | 1 | 1 | 1 | 1 | 1 | 1 | 1 | 0.5 | 1 | 1 | 10.5 |
| Kang Y et al. (2020) | 1 | 1 | 0.5 | 1 | 1 | 1 | 1 | 1 | 1 | 1 | 1 | 10.5 |
| Li H et al. (2020) | 1 | 0.5 | 1 | 1 | 1 | 1 | 1 | 1 | 0.5 | 1 | 1 | 10.0 |
| Lei W et al. (2015) | 1 | 1 | 1 | 1 | 1 | 1 | 1 | 1 | 1 | 1 | 1 | 11.0 |
| Miao Q et al. (2020) | 1 | 1 | 1 | 1 | 1 | 1 | 1 | 1 | 1 | 1 | 1 | 11.0 |
| Palaniyappan L et al. (2014) | 1 | 1 | 0.5 | 1 | 1 | 1 | 1 | 1 | 1 | 1 | 0.5 | 10.0 |
| Skåtun K et al. (2016) | 1 | 1 | 1 | 1 | 1 | 1 | 1 | 0.5 | 0.5 | 1 | 1 | 10.0 |
| Wang H et al. (2018) | 1 | 1 | 0.5 | 1 | 1 | 1 | 1 | 1 | 1 | 1 | 0.5 | 10.0 |
| Wang X et al. (2017) | 1 | 1 | 1 | 1 | 1 | 1 | 1 | 1 | 1 | 1 | 1 | 11.0 |
| Yang H et al. (2020) | 1 | 1 | 0.5 | 1 | 1 | 1 | 1 | 1 | 1 | 1 | 1 | 10.5 |
| Yu X et al. (2021) | 1 | 1 | 1 | 1 | 1 | 1 | 1 | 1 | 1 | 1 | 1 | 11.0 |
| Zhao J et al. (2022) | 1 | 1 | 1 | 1 | 1 | 1 | 1 | 1 | 0.5 | 1 | 1 | 10.5 |
| Zhou M et al. (2022) | 1 | 1 | 1 | 1 | 1 | 1 | 1 | 1 | 1 | 1 | 1 | 11.0 |
| Zhuo C et al. (2017) | 1 | 1 | 1 | 1 | 1 | 1 | 1 | 1 | 1 | 1 | 0.5 | 10.5 |

Table S3. The demographic information of each donor in AHBA dataset.

| **Donor ID** | **Ethnicity** | **Gender** | **Age** | **Hemisphere** |
| --- | --- | --- | --- | --- |
| 9861 | Black/African American | Male | 24 | L/R |
| 10021 | Black/African American | Male | 39 | L/R |
| 12876 | White/Caucasian | Male | 57 | L |
| 14380 | White/Caucasian | Male | 31 | L |
| 15496 | Hispanic | Female | 49 | L |
| 15697 | White/Caucasian | Male | 55 | L |

Abbreviations: AHBA, Allen Human Brain Atlas; ID, identification; L, left; R, right.

Table S4. Results of jackknife sensitivity analysis.

| **Discarded studies** | **Increased FNC** | | | **Decreased FNC** | | | | |
| --- | --- | --- | --- | --- | --- | --- | --- | --- |
|  | **Right IPC/SMG/ANG** | **Bilateral mPFC** | **Right DLPFC** | **Right insula** | **Left insula** | **Left PCG** | **Right PCG** | **Right ITG** |
| Chen C et al. (2015)^a^ | Yes | Yes | Yes | Yes | Yes | Yes | Yes | Yes |
| Chen C et al. (2015)^a^ | Yes | Yes | Yes | Yes | Yes | Yes | Yes | Yes |
| Chen J et al. (2022)^b^ | Yes | Yes | Yes | Yes | Yes | Yes | Yes | Yes |
| Chen J et al. (2022)^b^ | Yes | Yes | Yes | Yes | Yes | Yes | Yes | Yes |
| Chen X et al. (2018) | Yes | Yes | Yes | Yes | Yes | Yes | Yes | Yes |
| Chen X et al. (2019) | Yes | Yes | Yes | Yes | Yes | Yes | Yes | Yes |
| Chen X et al. (2019)^c^ | Yes | Yes | Yes | Yes | Yes | Yes | Yes | Yes |
| Chen X et al. (2019)^c^ | Yes | Yes | Yes | Yes | Yes | Yes | Yes | Yes |
| Ding Y et al. (2019) | Yes | Yes | Yes | Yes | Yes | Yes | Yes | Yes |
| Guo W et al. (2015) | Yes | Yes | Yes | Yes | Yes | Yes | Yes | Yes |
| Guo W et al. (2017) | Yes | Yes | Yes | Yes | Yes | Yes | Yes | Yes |
| Kang Y et al. (2020) | Yes | Yes | Yes | Yes | Yes | Yes | Yes | Yes |
| Li H et al. (2020) | Yes | Yes | Yes | Yes | Yes | Yes | Yes | Yes |
| Lei W et al. (2015) | Yes | Yes | Yes | Yes | Yes | Yes | Yes | Yes |
| Miao Q et al. (2020)^d^ | Yes | Yes | Yes | Yes | Yes | Yes | Yes | No |
| Miao Q et al. (2020)^d^ | Yes | Yes | Yes | Yes | Yes | Yes | Yes | No |
| Palaniyappan L et al. (2014) | Yes | Yes | Yes | Yes | Yes | Yes | No | Yes |
| Skåtun K et al. (2016) | Yes | Yes | No | Yes | Yes | Yes | Yes | Yes |
| Wang H et al. (2018) | Yes | Yes | Yes | Yes | Yes | Yes | Yes | Yes |
| Wang X et al. (2017) | Yes | Yes | Yes | Yes | Yes | Yes | No | Yes |
| Yang H et al. (2020) | Yes | Yes | Yes | Yes | Yes | Yes | Yes | Yes |
| Yu X et al. (2021) | Yes | Yes | Yes | Yes | Yes | Yes | Yes | Yes |
| Zhao J et al. (2022) | Yes | Yes | Yes | Yes | Yes | Yes | Yes | No |
| Zhou M et al. (2022) | Yes | Yes | Yes | Yes | Yes | Yes | Yes | Yes |
| Zhuo C et al. (2017) | Yes | Yes | Yes | Yes | Yes | No | No | No |
| **Total** | **25/25** | **25/25** | **24/25** | **25/25** | **25/25** | **24/25** | **22/25** | **21/25** |

The four studies (a-d) divided patients into 2 datasets based on different symptoms.

Abbreviations: ANG, angular gyrus; DLPFC, dorsolateral prefrontal cortex; FNC, functional network centrality; IPC, inferior parietal cortex; ITG, inferior temporal gyrus; mPFC, medial prefrontal cortex; PCG, postcentral gyrus; SMG, supramarginal gyrus.

Table S5. FNC changes in patients with schizophrenia in the subgroup analyses.

| **Regions** | **Peak MNI coordinates** | | | **Cluster size (voxels)** | **SDM-*Z* value** | ***P* value** |
| --- | --- | --- | --- | --- | --- | --- |
|  | *x* | ***y*** | ***z*** |  |  |  |
| **Drug naive/free: Increased FNCs** |  |  |  |  |  |  |
| Right angular gyrus, BA 40 | 56 | -50 | 34 | 2,052 | 2.219 | < 0.0001 |
| Left supplementary motor area, BA 6 | -6 | 18 | 64 | 556 | 1.968 | < 0.0001 |
| Cerebellum, vermic lobule VI | 2 | -72 | -18 | 15 | 1.329 | 0.0023 |
| **Drug naive/free: Decreased FNCs** |  |  |  |  |  |  |
| (undefined), BA 48 | 38 | -8 | -12 | 1,264 | -2.455 | < 0.0001 |
| Left cuneus cortex | -10 | -72 | 24 | 583 | -2.146 | < 0.0001 |
| Left superior temporal gyrus, BA 48 | -52 | -18 | 6 | 505 | -1.51 | 0.0021 |
| Right precentral gyrus, BA 44 | 36 | 2 | 32 | 33 | -1.678 | 0.0008 |
| **Drug used: Increased FNCs** |  |  |  |  |  |  |
| Left superior frontal gyrus, medial, BA 8 | -6 | 38 | 46 | 1,110 | 1.931 | < 0.0001 |
| Right olfactory cortex, BA 25 | 10 | 10 | -16 | 139 | 1.459 | 0.0019 |
| Left median network, cingulum | -26 | -24 | -20 | 91 | 1.456 | 0.002 |
| Left supplementary motor area, BA 6 | -6 | 0 | 68 | 52 | 1.45 | 0.002 |
| (undefined) | 8 | -26 | -4 | 46 | 1.456 | 0.002 |
| Left striatum | -8 | 2 | -8 | 37 | 1.455 | 0.002 |
| (undefined) | -6 | -28 | -2 | 35 | 1.447 | 0.002 |
| Left inferior temporal gyrus, BA 37 | -42 | -50 | -10 | 15 | 1.345 | 0.0038 |
| **Drug used: Decreased FNCs** |  |  |  |  |  |  |
| (undefined), BA 48 | 36 | -6 | 6 | 1,675 | -2.242 | < 0.0001 |
| Left postcentral gyrus, BA 4 | -60 | -6 | 40 | 1,000 | -1.95 | 0.0005 |
| (undefined), BA 48 | -32 | 2 | 4 | 617 | -1.881 | 0.0007 |
| Right precentral gyrus, BA 4 | 48 | -16 | 46 | 467 | -1.791 | 0.0012 |
| Right inferior temporal gyrus, BA 37 | 40 | -62 | -10 | 140 | -1.788 | 0.0012 |
| Right superior occipital gyrus, BA 18 | 22 | -92 | 14 | 136 | -1.727 | 0.0017 |
| Left cerebellum, hemispheric lobule VI, BA 18 | -18 | -74 | -14 | 127 | -1.786 | 0.0012 |
| Right cerebellum, hemispheric lobule VI, BA 37 | 28 | -54 | -24 | 24 | -1.606 | 0.0033 |
| **TR/TE (2000 ms/30 ms): Increased FNCs** |  |  |  |  |  |  |
| Right inferior parietal (excluding supramarginal and angular) gyri, BA 40 | 54 | -52 | 38 | 1,974 | 2.502 | < 0.0001 |
| Left superior frontal gyrus, medial, BA 8 | -6 | 38 | 44 | 1,710 | 2.507 | < 0.0001 |
| Right precuneus | 8 | -60 | 38 | 763 | 1.82 | 0.0002 |
| **TR/TE (2000 ms/30 ms): Decreased FNCs** |  |  |  |  |  |  |
| (undefined), BA 48 | 38 | -6 | -10 | 2,927 | -3.408 | < 0.0001 |
| Left lenticular nucleus, putamen, BA 48 | -30 | -4 | 0 | 1,857 | -2.059 | < 0.0001 |
| Right precentral gyrus, BA 44 | 36 | 2 | 32 | 14 | -1.523 | 0.0022 |
| **GE: Increased FNCs** |  |  |  |  |  |  |
| Left superior frontal gyrus, dorsolateral, BA 9 | -12 | 46 | 40 | 772 | 1.543 | 0.0003 |
| Right superior frontal gyrus, dorsolateral, BA 9 | 18 | 42 | 50 | 163 | 1.417 | 0.001 |
| Left hippocampus, BA 20 | -26 | -22 | -16 | 86 | 1.327 | 0.0019 |
| (undefined), BA 25 | 12 | 2 | -12 | 75 | 1.328 | 0.0019 |
| Right thalamus | 12 | -26 | 0 | 49 | 1.328 | 0.0019 |
| Left supplementary motor area, BA 6 | -8 | 0 | 70 | 49 | 1.327 | 0.0019 |
| Cerebellum, vermic lobule VI | 4 | -72 | -18 | 46 | 1.438 | 0.0008 |
| Left anterior thalamic projections | -10 | 4 | -2 | 36 | 1.327 | 0.0019 |
| Left thalamus | -8 | -28 | 0 | 34 | 1.323 | 0.002 |
| Left inferior temporal gyrus, BA 37 | -42 | -50 | -10 | 14 | 1.324 | 0.002 |
| **GE: Decreased FNCs** |  |  |  |  |  |  |
| (undefined), BA 48 | 36 | -6 | -12 | 2,375 | -2.947 | < 0.0001 |
| Left lenticular nucleus, putamen | -28 | 0 | 6 | 303 | -1.583 | 0.0023 |
| Right inferior temporal gyrus, BA 37 | 40 | -62 | -12 | 134 | -1.622 | 0.0019 |
| Right cerebellum, hemispheric lobule VI, BA 37 | 26 | -54 | -22 | 36 | -1.538 | 0.0028 |
| Left precentral gyrus | -52 | -2 | 38 | 21 | -1.47 | 0.0042 |
| Left postcentral gyrus, BA 4 | -50 | -20 | 42 | 19 | -1.467 | 0.0042 |
| Right inferior frontal gyrus, opercular part, BA 6 | 36 | 4 | 32 | 11 | -1.475 | 0.0041 |
| **Siemens: Increased FNCs** |  |  |  |  |  |  |
| Right angular gyrus, BA 39 | 52 | -58 | 42 | 2,089 | 2.361 | < 0.0001 |
| Left supplementary motor area, BA 6 | -6 | 16 | 64 | 1,005 | 2.477 | < 0.0001 |
| Right precuneus | 6 | -64 | 44 | 157 | 1.988 | < 0.0001 |
| Right middle frontal gyrus, BA 9 | 42 | 24 | 44 | 21 | 1.389 | 0.0034 |
| **Siemens: Decreased FNCs** |  |  |  |  |  |  |
| Left superior temporal gyrus, BA 48 | -52 | -18 | 6 | 1,409 | -1.526 | 0.0007 |
| Left calcarine fissure / surrounding cortex, BA 17 | 2 | -80 | 12 | 238 | -1.483 | 0.0011 |
| Right inferior temporal gyrus, BA 20 | 58 | -46 | -16 | 15 | -1.318 | 0.0033 |
| **FWHM (6 mm): Increased FNCs** |  |  |  |  |  |  |
| Left superior frontal gyrus, medial, BA 8 | -6 | 38 | 44 | 831 | 1.79 | < 0.0001 |
| Left cerebellum, crus I | -44 | -52 | -36 | 532 | 1.495 | 0.001 |
| Right superior frontal gyrus, dorsolateral, BA 8 | 10 | 32 | 50 | 120 | 1.436 | 0.0016 |
| Right inferior parietal (excluding supramarginal and angular) gyri | 52 | -58 | 48 | 69 | 1.466 | 0.0013 |
| Cerebellum, vermic lobule VI | 2 | -72 | -18 | 35 | 1.427 | 0.0017 |
| Right pons | 10 | -22 | -4 | 29 | 1.314 | 0.0033 |
| Right striatum | 14 | 2 | -10 | 26 | 1.312 | 0.0033 |
| Left striatum | -8 | 2 | -10 | 21 | 1.314 | 0.0033 |
| Left hippocampus, BA 37 | -26 | -30 | -8 | 21 | 1.311 | 0.0034 |
| Left supplementary motor area, BA 6 | -6 | -2 | 68 | 21 | 1.308 | 0.0034 |
| Left hippocampus, BA 20 | -28 | -26 | -14 | 17 | 1.307 | 0.0035 |
| Left thalamus | -16 | -20 | 18 | 13 | 1.295 | 0.0038 |
| Right striatum | 8 | 14 | -6 | 12 | 1.313 | 0.0033 |
| **FWHM (6 mm): Decreased FNCs** |  |  |  |  |  |  |
| Right insula, BA 48 | 34 | -6 | -12 | 2,123 | -2.8 | < 0.0001 |
| Left postcentral gyrus, BA 43 | -58 | -8 | 26 | 736 | -1.568 | 0.0025 |
| Left lenticular nucleus, putamen, BA 48 | -30 | 8 | 4 | 565 | -1.797 | 0.0005 |
| Right postcentral gyrus, BA 4 | 46 | -18 | 50 | 244 | -1.573 | 0.0024 |
| Right superior occipital gyrus, BA 18 | 14 | -84 | 12 | 114 | -1.609 | 0.002 |
| **Global signal not removed: Increased FNCs** |  |  |  |  |  |  |
| Right inferior parietal (excluding supramarginal and angular) gyri, BA 40 | 54 | -54 | 38 | 1,603 | 2.436 | < 0.0001 |
| Left supplementary motor area, BA 6 | -6 | 18 | 60 | 734 | 2.231 | < 0.0001 |
| Right superior frontal gyrus, dorsolateral, BA 8 | 26 | 26 | 50 | 157 | 1.705 | 0.0007 |
| Right striatum | 10 | 10 | -6 | 87 | 1.485 | 0.0024 |
| Right precuneus | 6 | -60 | 42 | 58 | 1.592 | 0.0014 |
| Left striatum | -12 | 6 | -6 | 33 | 1.477 | 0.0025 |
| **Global signal not removed: Decreased FNCs** |  |  |  |  |  |  |
| Left postcentral gyrus, BA 4 | -54 | -12 | 26 | 1,828 | -1.955 | 0.0002 |
| Right inferior temporal gyrus, BA 37 | 46 | -64 | -10 | 369 | -2.087 | < 0.0001 |
| Right cuneus cortex, BA 18 | 16 | -86 | 12 | 145 | -1.748 | 0.001 |
| Right postcentral gyrus, BA 4 | 48 | -20 | 46 | 77 | -1.537 | 0.0028 |
| Right middle frontal gyrus, BA 46 | 28 | 36 | 28 | 26 | -1.563 | 0.0025 |
| Right middle frontal gyrus | 26 | 60 | 28 | 10 | -1.582 | 0.0023 |
| **Binary FNC: Increased FNCs** |  |  |  |  |  |  |
| Left cerebellum, crus I | -44 | -52 | -36 | 736 | 1.533 | 0.0006 |
| Left supplementary motor area, BA 6 | -4 | 6 | 66 | 240 | 1.757 | 0.0001 |
| Right inferior parietal (excluding supramarginal and angular) gyri | 52 | -58 | 48 | 166 | 1.503 | 0.0008 |
| Right superior frontal gyrus, dorsolateral, BA 8 | 10 | 32 | 50 | 156 | 1.476 | 0.001 |
| Right striatum | 16 | 18 | -12 | 105 | 1.294 | 0.003 |
| Left anterior thalamic projections | -8 | 2 | -4 | 76 | 1.294 | 0.003 |
| Left hippocampus, BA 20 | -24 | -32 | -8 | 65 | 1.294 | 0.003 |
| (undefined) | 8 | -26 | -6 | 43 | 1.295 | 0.003 |
| Left thalamus | -12 | -18 | 18 | 39 | 1.295 | 0.003 |
| Left thalamus | -8 | -28 | 0 | 33 | 1.287 | 0.0031 |
| **Binary FNC: Decreased FNCs** |  |  |  |  |  |  |
| Left postcentral gyrus, BA 4 | -50 | -12 | 40 | 1,295 | -1.941 | 0.0001 |
| Right precentral gyrus, BA 4 | 44 | -18 | 44 | 942 | -1.864 | 0.0003 |
| Right superior occipital gyrus, BA 18 | 16 | -84 | 16 | 150 | -1.603 | 0.0019 |
| Right striatum | 30 | -10 | -4 | 132 | -1.549 | 0.0025 |
| Right inferior frontal gyrus, opercular part, BA 6 | 38 | 4 | 34 | 11 | -1.512 | 0.0031 |
| **Weighted FNC: Increased FNCs** |  |  |  |  |  |  |
| Left superior frontal gyrus, medial, BA 9 | -6 | 40 | 46 | 1,754 | 2.629 | < 0.0001 |
| Right inferior parietal (excluding supramarginal and angular) gyri, BA 40 | 54 | -54 | 44 | 1,582 | 1.903 | < 0.0001 |
| Left precuneus | 0 | -58 | 42 | 820 | 1.683 | 0.0003 |
| **Weighted FNC: Decreased FNCs** |  |  |  |  |  |  |
| (undefined), BA 48 | 36 | -6 | -10 | 2,702 | -2.917 | < 0.0001 |
| Left lenticular nucleus, putamen, BA 48 | -30 | 0 | 2 | 891 | -2.215 | < 0.0001 |
| Right fusiform gyrus, BA 37 | 42 | -60 | -18 | 534 | -1.903 | 0.0004 |
| Right superior frontal gyrus, medial orbital, BA 11 | 6 | 62 | -14 | 210 | -1.704 | 0.0012 |

Table S6. Genes that are positively and negatively weighted on PLS2.

| Positively weighted genes | Z score | Negatively weighted genes | Z score |
| --- | --- | --- | --- |
| *SPHKAP* | 9.487594 | *MPP7* | -10.8974 |
| *KCTD12* | 9.234364 | *FBXO40* | -10.8971 |
| *EPHX1* | 9.159512 | *PTGS2* | -10.464 |
| *ZIC1* | 8.777558 | *FMN1* | -10.3252 |
| *KIAA0319* | 8.677402 | *MCHR2* | -10.1188 |
| *KLF12* | 8.609615 | *CD52* | -10.0156 |
| *ARG2* | 8.609048 | *LINC00622* | -9.96031 |
| *SLC24A3* | 8.605433 | *SLC17A8* | -9.94835 |
| *TANC1* | 8.515325 | *SMIM32* | -9.5778 |
| *CELF2* | 8.203407 | *TMEM196* | -9.41854 |
| *CTNNB1* | 7.950211 | *ISLR* | -9.40972 |
| *FZD1* | 7.918817 | *TMEM132D* | -9.40793 |
| *KCNB2* | 7.812844 | *THEMIS* | -9.35816 |
| *PLPP4* | 7.78162 | *ITGA11* | -9.35572 |
| *SUSD4* | 7.766011 | *MTCL1* | -9.25554 |
| *GDF10* | 7.707028 | *CTNNAL1* | -9.24976 |
| *HECA* | 7.70492 | *CUX1* | -9.24575 |
| *SORL1* | 7.657255 | *ARPP19* | -9.13276 |
| *DOK7* | 7.637317 | *ART3* | -9.06096 |
| *ENAH* | 7.633405 | *NR4A2* | -8.97073 |
| *SYNGR1* | 7.59693 | *SLCO2A1* | -8.9157 |
| *NRP1* | 7.456591 | *HSPB3* | -8.85955 |
| *EPHA6* | 7.45086 | *FAP* | -8.85292 |
| *CARTPT* | 7.411985 | *MADCAM1* | -8.85004 |
| *DECR1* | 7.380905 | *KIRREL2* | -8.8329 |
| *PTPRZ1* | 7.340984 | *STAC2* | -8.786 |
| *CEP112* | 7.291429 | *PLA2G4A* | -8.76669 |
| *RASL12* | 7.267782 | *GCNT4* | -8.76667 |
| *DUSP4* | 7.234573 | *GPR6* | -8.71372 |
| *KIT* | 7.233962 | *ANXA1* | -8.6873 |
| *MYT1* | 7.214124 | *OPN4* | -8.68247 |
| *FAM19A5* | 7.20305 | *ZNF697* | -8.66333 |
| *CRNDE* | 7.177462 | *TC2N* | -8.57359 |
| *CDC42EP4* | 7.165027 | *NPY1R* | -8.52119 |
| *PAG1* | 7.155692 | *DRD1* | -8.5194 |
| *MGST1* | 7.14567 | *TBR1* | -8.50926 |
| *FAM20A* | 7.103941 | *SYT10* | -8.49 |
| *ADCYAP1R1* | 7.085778 | *KCTD16* | -8.4767 |
| *CDS1* | 7.079539 | *TESPA1* | -8.44893 |
| *DPY19L3* | 7.007282 | *FOSB* | -8.41887 |
| *TSPAN11* | 6.967821 | *LOC100129291* | -8.37446 |
| *RAVER2* | 6.924619 | *RSPO2* | -8.36769 |
| *SMCO4* | 6.897763 | *CDKL2* | -8.34382 |
| *FGFR1* | 6.893548 | *PCDH11Y* | -8.32275 |
| *DCXR* | 6.877414 | *RPS6KA2* | -8.32145 |
| *TENM1* | 6.85546 | *RXFP1* | -8.24233 |
| *LRCH1* | 6.835105 | *UNC5B-AS1* | -8.24052 |
| *CHRNA3* | 6.82341 | *TNNT2* | -8.22915 |
| *ST8SIA1* | 6.821708 | *KRT14* | -8.20952 |
| *NTPCR* | 6.80135 | *ISLR2* | -8.15018 |
| *CTDSP2* | 6.796763 | *SCGB3A1* | -8.14547 |
| *TEAD1* | 6.791789 | *GRASP* | -8.13775 |
| *KLHL5* | 6.784917 | *NRN1L* | -8.00009 |
| *CHST8* | 6.761647 | *TSPAN13* | -7.97661 |
| *ADRA1D* | 6.737396 | *OVOL2* | -7.97317 |
| *WNT4* | 6.712359 | *ADRA2C* | -7.95938 |
| *NHSL1* | 6.648361 | *PP12613* | -7.958 |
| *NRIP3* | 6.642866 | *L1TD1* | -7.93424 |
| *PDLIM3* | 6.610634 | *EFNA5* | -7.91165 |
| *ZIC4* | 6.59624 | *KCNS2* | -7.89947 |
| *UST* | 6.593204 | *CNST* | -7.89312 |
| *PCCA* | 6.565926 | *EPB41L1* | -7.89056 |
| *TMEM229A* | 6.560987 | *LOC100507351* | -7.88075 |
| *KLHDC8A* | 6.556266 | *TAC3* | -7.87162 |
| *HVCN1* | 6.537168 | *LCE3C* | -7.82457 |
| *TCF7L2* | 6.481386 | *TMEM200A* | -7.71506 |
| *ROBO1* | 6.479664 | *KANSL3* | -7.6974 |
| *DDAH1* | 6.475402 | *UNC5D* | -7.64966 |
| *SHISA6* | 6.471198 | *PART1* | -7.62524 |
| *GYG2* | 6.426023 | *DUSP23* | -7.61779 |
| *SULT1C4* | 6.408187 | *MALL* | -7.61258 |
| *BAALC* | 6.398543 | *NWD2* | -7.60598 |
| *NEFH* | 6.384518 | *NEURL3* | -7.57366 |
| *PTN* | 6.378855 | *RGS12* | -7.56983 |
| *RNF182* | 6.375423 | *PRSS35* | -7.56402 |
| *CALB2* | 6.351522 | *NPPA* | -7.55518 |
| *ASCL1* | 6.343323 | *SLC24A4* | -7.54792 |
| *MID1* | 6.341708 | *CFD* | -7.54304 |
| *LATS2* | 6.334568 | *LINC01102* | -7.49464 |
| *HIST1H4C* | 6.334425 | *CIDEA* | -7.49385 |
| *PREB* | 6.321925 | *LOC101928087* | -7.45898 |
| *CTNNBIP1* | 6.290263 | *GABRE* | -7.4307 |
| *SEC61A1* | 6.290117 | *TPMT* | -7.39774 |
| *QPCT* | 6.269309 | *AVPI1* | -7.38927 |
| *SH3PXD2B* | 6.24638 | *CD163L1* | -7.36589 |
| *DNAH14* | 6.234302 | *KIDINS220* | -7.35714 |
| *MYBPC1* | 6.225734 | *AKAP1* | -7.35155 |
| *JAK1* | 6.222332 | *TMEM233* | -7.33508 |
| *USP6NL* | 6.219702 | *SLN* | -7.32999 |
| *CAMK2D* | 6.211914 | *NPHS1* | -7.32718 |
| *ATP1B1* | 6.19993 | *ACKR1* | -7.31594 |
| *LAMA1* | 6.199409 | *ZMIZ1* | -7.28954 |
| *WBP1L* | 6.194202 | *OSTN* | -7.28445 |
| *CHST9* | 6.194048 | *STT3B* | -7.28158 |
| *SH3KBP1* | 6.189249 | *TCAP* | -7.27923 |
| *CTIF* | 6.181291 | *EIF4E1B* | -7.2781 |
| *CCDC80* | 6.166388 | *BCL2L11* | -7.26436 |
| *PCDHB5* | 6.165982 | *ARHGAP9* | -7.24774 |
| *PARD3* | 6.165117 | *LY86-AS1* | -7.24015 |
| *ANKRD34C* | 6.152678 | *CYP26A1* | -7.2349 |
| *FBXL4* | 6.15164 | *HGF* | -7.22615 |
| *SLC35D1* | 6.146827 | *CABP1* | -7.21632 |
| *NLGN1* | 6.145 | *SLC22A9* | -7.20811 |
| *PRRX1* | 6.137883 | *ADGRL2* | -7.20327 |
| *FANK1* | 6.13715 | *GALNTL5* | -7.17317 |
| *ERICH1* | 6.122042 | *GNA14* | -7.16051 |
| *OPRM1* | 6.119843 | *ANTXR2* | -7.15975 |
| *EDNRB* | 6.094771 | *EGR3* | -7.15611 |
| *ADCY8* | 6.084064 | *LYPD8* | -7.1003 |
| *PTPN13* | 6.074553 | *GABRG3* | -7.08959 |
| *XYLT2* | 6.057866 | *PIF1* | -7.08496 |
| *ADIRF* | 6.054126 | *STYK1* | -7.08395 |
| *C2CD2* | 6.047331 | *VIPR1* | -7.06556 |
| *MIPEP* | 6.038508 | *PALM3* | -7.03079 |
| *ALKBH6* | 6.037847 | *TNNC2* | -7.03045 |
| *GULP1* | 6.036229 | *KCNS1* | -7.02675 |
| *MSRA* | 6.028347 | *DLGAP2* | -7.01235 |
| *ZNF608* | 6.025886 | *ATP4A* | -7.01208 |
| *EPHX2* | 6.012862 | *FAM19A1* | -7.00504 |
| *ALDOC* | 6.012263 | *LINC00958* | -6.99998 |
| *ZNF653* | 5.996271 | *DENND3* | -6.99587 |
| *KIRREL3* | 5.994585 | *GFPT2* | -6.98788 |
| *C14orf132* | 5.98914 | *DLGAP1-AS4* | -6.98618 |
| *SHC1* | 5.976887 | *LINC00507* | -6.97946 |
| *TMEM50B* | 5.967225 | *HERC6* | -6.97115 |
| *TTC9* | 5.963873 | *RASGRF2* | -6.96719 |
| *MAGED2* | 5.962064 | *TMEM155* | -6.95598 |
| *RASGRF1* | 5.943742 | *FAM71F1* | -6.93707 |
| *COL9A1* | 5.941521 | *SLC26A4* | -6.92665 |
| *KRT31* | 5.936922 | *PIGK* | -6.89171 |
| *DHRS4* | 5.903917 | *CEMIP* | -6.88842 |
| *ZNF219* | 5.893019 | *LINC01551* | -6.86796 |
| *BCL7C* | 5.889377 | *EPOP* | -6.83564 |
| *CHCHD6* | 5.886637 | *CACNA1E* | -6.82498 |
| *LRRC37A4P* | 5.877524 | *FLJ33534* | -6.78863 |
| *PCK2* | 5.876699 | *ADTRP* | -6.78745 |
| *ATP13A2* | 5.870249 | *IER5* | -6.77363 |
| *AQP1* | 5.867567 | *OCA2* | -6.76989 |
| *SPHK2* | 5.858483 | *SEMA6D* | -6.76641 |
| *NOTCH2NL* | 5.856409 | *FOXG1* | -6.74758 |
| *NYAP2* | 5.850515 | *ARHGAP32* | -6.74589 |
| *EIF4G2* | 5.846714 | *NOS2* | -6.73784 |
| *H3F3B* | 5.844504 | *HS3ST4* | -6.73416 |
| *ITGA6* | 5.842091 | *HTR1A* | -6.71057 |
| *PDE3A* | 5.834155 | *ARL4A* | -6.7103 |
| *COL11A1* | 5.820625 | *ZNF385D* | -6.70711 |
| *FAM89A* | 5.818454 | *KCNH1* | -6.70586 |
| *PTAR1* | 5.812131 | *RILPL1* | -6.68922 |
| *AKR7A3* | 5.798567 | *SATB2* | -6.67956 |
| *FAAP20* | 5.793415 | *GNB4* | -6.66952 |
| *RPS27L* | 5.786705 | *EPB41L4A* | -6.66028 |
| *IGDCC3* | 5.785358 | *ALCAM* | -6.65705 |
| *NPTX2* | 5.784143 | *LOC440040* | -6.65308 |
| *FNDC5* | 5.779898 | *PLEKHA1* | -6.65121 |
| *CERS1* | 5.777268 | *ADCY2* | -6.64531 |
| *FAM182B* | 5.772388 | *JPH1* | -6.64079 |
| *ZBTB20* | 5.761677 | *CRLF1* | -6.62262 |
| *TIMP3* | 5.756475 | *OPRK1* | -6.61959 |
| *FGD4* | 5.753694 | *KLHL1* | -6.61896 |
| *FAM210B* | 5.751786 | *LXN* | -6.61553 |
| *PGAP1* | 5.749967 | *PDGFD* | -6.61451 |
| *ZNF260* | 5.746764 | *RGS4* | -6.61313 |
| *HS6ST2* | 5.737986 | *KANK4* | -6.59482 |
| *MRPS23* | 5.736404 | *KCNG3* | -6.58872 |
| *SOX11* | 5.724296 | *SOSTDC1* | -6.58629 |
| *PACSIN2* | 5.719959 | *TMEM145* | -6.57531 |
| *CHRNA4* | 5.713403 | *WNT2B* | -6.57456 |
| *ST5* | 5.710148 | *VIP* | -6.55683 |
| *SRRM4* | 5.708116 | *TCIM* | -6.5499 |
| *RBKS* | 5.706986 | *ANKMY2* | -6.53646 |
| *PHGDH* | 5.691526 | *SLC39A4* | -6.52039 |
| *MCRIP2* | 5.686896 | *PRDM8* | -6.50511 |
| *DOC2B* | 5.685953 | *KCNV1* | -6.5024 |
| *RAB8B* | 5.685564 | *CDH12* | -6.49775 |
| *GLUD1* | 5.682643 | *RASGEF1C* | -6.47519 |
| *ABCG4* | 5.681012 | *SOHLH1* | -6.45535 |
| *ZNF124* | 5.679656 | *CRYBB1* | -6.45216 |
| *JKAMP* | 5.671583 | *ADCY7* | -6.45069 |
| *MINOS1* | 5.659325 | *MEDAG* | -6.42822 |
| *CETN3* | 5.653352 | *PTGER3* | -6.42658 |
| *LNX2* | 5.65189 | *PAOX* | -6.42177 |
| *PIP4P2* | 5.648614 | *MEF2C* | -6.42039 |
| *NUPR1* | 5.644379 | *GLA* | -6.41835 |
| *ERI3* | 5.632371 | *RMI2* | -6.41141 |
| *MAPKAPK2* | 5.625159 | *PECAM1* | -6.39163 |
| *BPHL* | 5.617404 | *LAMB3* | -6.34916 |
| *PHB* | 5.605743 | *CCDC3* | -6.34176 |
| *PDGFRA* | 5.605184 | *KLHL4* | -6.3402 |
| *GNA12* | 5.593894 | *NRAP* | -6.3016 |
| *PPP1R14B* | 5.57775 | *DLX1* | -6.27993 |
| *TGFBR1* | 5.57768 | *KRT17* | -6.27133 |
| *CPNE2* | 5.57412 | *PCDH11X* | -6.26894 |
| *MLLT1* | 5.566605 | *PSORS1C1* | -6.26384 |
| *RND2* | 5.560991 | *TLE2* | -6.25438 |
| *IGLON5* | 5.556269 | *RPRML* | -6.24745 |
| *NEURL1B* | 5.5478 | *ZNF831* | -6.247 |
| *EML4* | 5.545538 | *ZBTB8A* | -6.23651 |
| *TRPM3* | 5.544882 | *MGAT5* | -6.22621 |
| *SLC14A1* | 5.53914 | *HBQ1* | -6.22401 |
| *CCDC151* | 5.53788 | *CHSY3* | -6.215 |
| *C2orf40* | 5.537528 | *GPR88* | -6.21075 |
| *SPOCK2* | 5.53012 | *ABCC8* | -6.20912 |
| *KCNA5* | 5.506196 | *SMIM27* | -6.20365 |
| *IDH1* | 5.506077 | *FAM131B* | -6.2006 |
| *TFB1M* | 5.499888 | *NUAK1* | -6.19077 |
| *ASXL2* | 5.499759 | *EPHX4* | -6.18217 |
| *PITPNM1* | 5.499285 | *C2CD4C* | -6.17861 |
| *TMCC2* | 5.496294 | *TRAM2* | -6.15603 |
| *SRM* | 5.490469 | *PRKCB* | -6.14834 |
| *FBXW4* | 5.489719 | *TSPYL1* | -6.14704 |
| *FUNDC2* | 5.485524 | *LOC105376360* | -6.14677 |
| *ZBTB33* | 5.484047 | *LOR* | -6.13853 |
| *NDN* | 5.480303 | *LRRC4C* | -6.13495 |
| *FAM43B* | 5.478686 | *VIT* | -6.13441 |
| *TMTC1* | 5.455513 | *PASK* | -6.13344 |
| *FH* | 5.454206 | *FZD3* | -6.12551 |
| *VCAN* | 5.45295 | *OR2L13* | -6.12331 |
| *HBB* | 5.446194 | *CADPS2* | -6.12282 |
| *DPCD* | 5.433801 | *STARD5* | -6.11941 |
| *METRN* | 5.428555 | *NTSR1* | -6.11741 |
| *SRGAP2* | 5.425373 | *ZNRF4* | -6.09195 |
| *WLS* | 5.420816 | *KLF10* | -6.09042 |
| *PLOD1* | 5.418333 | *ERC2-IT1* | -6.08524 |
| *STK26* | 5.393839 | *POU2F2* | -6.0844 |
| *RNF185* | 5.393413 | *TAGLN2* | -6.08281 |
| *EPHA4* | 5.389533 | *OSCAR* | -6.08251 |
| *ENKUR* | 5.388841 | *IER5L* | -6.07909 |
| *ZRSR2* | 5.387906 | *LRRC39* | -6.07821 |
| *GPRIN2* | 5.384147 | *FAM212B* | -6.07678 |
| *HDC* | 5.376535 | *EGR2* | -6.07464 |
| *NEK11* | 5.372623 | *CRSP8P* | -6.06972 |
| *BCL2* | 5.363554 | *WDHD1* | -6.05974 |
| *ZC3H11B* | 5.357255 | *SLITRK1* | -6.0573 |
| *PRKCA* | 5.347947 | *TMEM86B* | -6.05446 |
| *MPZL3* | 5.346349 | *KCNQ5* | -6.0543 |
| *HOMER3* | 5.340263 | *PNMT* | -6.05313 |
| *WASHC1* | 5.33773 | *CHRD* | -6.04085 |
| *SYT9* | 5.33685 | *FOSL2* | -6.03791 |
| *TRPT1* | 5.333842 | *ASB5* | -6.03342 |
| *HSPB8* | 5.33349 | *GRIP1* | -6.03214 |
| *INPP5J* | 5.325869 | *ABLIM2* | -6.02185 |
| *RBP1* | 5.324578 | *PHYHIPL* | -5.9971 |
| *CSDC2* | 5.305724 | *SLC4A11* | -5.99648 |
| *PDCL3P4* | 5.301906 | *LINC01106* | -5.98669 |
| *C11orf49* | 5.292132 | *IFT172* | -5.98272 |
| *PCDH19* | 5.285674 | *NPY* | -5.9782 |
| *ST6GALNAC5* | 5.280136 | *SETBP1* | -5.97145 |
| *CTSH* | 5.27614 | *SHISA9* | -5.96246 |
| *CRISPLD1* | 5.263536 | *A1BG* | -5.9425 |
| *CAMK2N2* | 5.26078 | *COL13A1* | -5.94204 |
| *FABP5* | 5.252392 | *CAP1* | -5.94006 |
| *C2orf72* | 5.248725 | *LSM3* | -5.93699 |
| *BCHE* | 5.245194 | *BATF3* | -5.93207 |
| *HSBP1L1* | 5.234116 | *TCHH* | -5.92218 |
| *GOLGA2P7* | 5.232863 | *PRSS3P2* | -5.9136 |
| *ARHGEF40* | 5.22833 | *RAB12* | -5.90808 |
| *MYL6* | 5.223378 | *MORN3* | -5.89835 |
| *TMEM164* | 5.215672 | *NPY5R* | -5.89107 |
| *UNKL* | 5.215371 | *DACH1* | -5.88403 |
| *FAM86EP* | 5.211112 | *B3GNT4* | -5.88009 |
| *ZNF610* | 5.210714 | *CHRM1* | -5.87766 |
| *MAFK* | 5.202445 | *LMO3* | -5.87535 |
| *PDE12* | 5.195149 | *NTNG2* | -5.86279 |
| *HMGN5* | 5.191109 | *SYDE2* | -5.8616 |
| *ERC1* | 5.190714 | *AMZ1* | -5.86051 |
| *C6orf118* | 5.188209 | *RPS6KA3* | -5.85851 |
| *IPW* | 5.183999 | *C19orf66* | -5.85757 |
| *TCTEX1D1* | 5.183569 | *SACS* | -5.85688 |
| *NTSR2* | 5.180647 | *LINC00663* | -5.85515 |
| *GPR26* | 5.179419 | *LINC01140* | -5.84348 |
| *NCOA1* | 5.176819 | *DONSON* | -5.83932 |
| *CD24* | 5.176589 | *CRHBP* | -5.83873 |
| *CARMIL3* | 5.17276 | *MYOM2* | -5.83728 |
| *CUTC* | 5.166732 | *HLF* | -5.83544 |
| *CD83* | 5.156645 | *CCKBR* | -5.82304 |
| *FDX1* | 5.155537 | *NETO1* | -5.81274 |
| *RCC2* | 5.152264 | *FEZF2* | -5.79397 |
| *HSDL2* | 5.14771 | *LSP1* | -5.78863 |
| *OCRL* | 5.14734 | *DRP2* | -5.78045 |
| *SNX10* | 5.145276 | *SLC26A4-AS1* | -5.76832 |
| *TCERG1L* | 5.13719 | *RRAS* | -5.76148 |
| *CASK* | 5.135179 | *IGSF23* | -5.76051 |
| *ABRACL* | 5.113752 | *PTPRT* | -5.75597 |
| *RPS19BP1* | 5.11177 | *LOC729683* | -5.75288 |
| *NANS* | 5.1111 | *COX7A1* | -5.75266 |
| *GHR* | 5.110575 | *HGD* | -5.74826 |
| *IGSF1* | 5.090216 | *OR14I1* | -5.74297 |
| *ELMOD2* | 5.086658 | *HDGFL1* | -5.74293 |
| *ACADVL* | 5.079883 | *MPP1* | -5.73047 |
| *UBE2F* | 5.075074 | *SEMA3A* | -5.72862 |
| *WIPF3* | 5.074181 | *TTN* | -5.72227 |
| *LRRTM1* | 5.073593 | *UBQLN2* | -5.70854 |
| *BRD3OS* | 5.072667 | *LRRC38* | -5.68322 |
| *DYRK2* | 5.0723 | *FOXP1* | -5.68172 |
| *MAP3K20* | 5.071954 | *DDB2* | -5.68047 |
| *TMEM229B* | 5.070097 | *CFP* | -5.67883 |
| *ARMCX3* | 5.069122 | *ARAP2* | -5.67618 |
| *FAM3A* | 5.06752 | *LTK* | -5.66537 |
| *EPHA1* | 5.065416 | *EXO5* | -5.65346 |
| *CORO2A* | 5.061574 | *RAD54L* | -5.65335 |
| *CA12* | 5.05671 | *SKAP2* | -5.64818 |
| *S100PBP* | 5.055841 | *RTKN2* | -5.64146 |
| *BDH2* | 5.055313 | *PPP1R1A* | -5.63906 |
| *NECTIN1* | 5.055124 | *TRPC4* | -5.63699 |
| *SESN2* | 5.049443 | *GSG1L* | -5.63627 |
| *NFIX* | 5.045811 | *TMEM255B* | -5.62956 |
| *FAM86FP* | 5.040016 | *ANAPC5* | -5.62915 |
| *RAB27B* | 5.038689 | *SLIT1* | -5.62849 |
| *RNF115* | 5.038354 | *RASL11B* | -5.62628 |
| *C9orf147* | 5.030778 | *OSGEP* | -5.61974 |
| *NTM* | 5.028732 | *ADGRV1* | -5.61817 |
| *AKAP12* | 5.021417 | *KLF7* | -5.60744 |
| *PDE5A* | 5.021188 | *GLIS1* | -5.60042 |
| *DPP6* | 5.012893 | *NEUROD6* | -5.59772 |
| *NKAIN4* | 5.007175 | *GTDC1* | -5.59321 |
| *PLA2G5* | 4.999657 | *PRSS2* | -5.58809 |
| *BFAR* | 4.999311 | *TYRP1* | -5.56446 |
| *TOX2* | 4.995845 | *MBD2* | -5.56249 |
| *TSPAN18* | 4.984564 | *CSE1L* | -5.56213 |
| *IMPAD1* | 4.980402 | *COMTD1* | -5.5575 |
| *THSD4* | 4.975611 | *CUX2* | -5.55455 |
| *PRMT1* | 4.975003 | *PARP2* | -5.54507 |
| *UNC119B* | 4.973332 | *TPTE2P6* | -5.54326 |
| *LOC105370333* | 4.966703 | *JAK2* | -5.53843 |
| *SEC11C* | 4.965785 | *GNG2* | -5.53767 |
| *SEMA3E* | 4.965224 | *COL19A1* | -5.5333 |
| *CLU* | 4.96479 | *MPPED1* | -5.5288 |
| *IL1RAP* | 4.963971 | *BLOC1S1* | -5.52552 |
| *UBE2L6* | 4.961862 | *SNRNP27* | -5.52438 |
| *IDH2* | 4.959387 | *ANXA11* | -5.51736 |
| *ICAM3* | 4.955756 | *PTBP3* | -5.51336 |
| *SYT3* | 4.952701 | *RAB7B* | -5.51207 |
| *RSPO4* | 4.946957 | *FAM161A* | -5.50377 |
| *HIST1H4L* | 4.932796 | *ADAM22* | -5.50177 |
| *GLIPR1* | 4.930308 | *ANO7* | -5.4991 |
| *GREM2* | 4.928397 | *GAST* | -5.49888 |
| *RELA* | 4.928236 | *DLX2* | -5.49745 |
| *PGD* | 4.927096 | *SENP6* | -5.49372 |
| *DND1* | 4.921081 | *SYT16* | -5.4886 |
| *FAM110A* | 4.920348 | *GDPD5* | -5.48328 |
| *TRMT12* | 4.917253 | *KCNH4* | -5.47486 |
| *CMTM3* | 4.913778 | *KCNT1* | -5.47345 |
| *ADI1* | 4.912614 | *ADAMTS9* | -5.46577 |
| *C3orf70* | 4.907778 | *SOWAHB* | -5.46568 |
| *KIAA1211* | 4.907192 | *LRRTM4* | -5.46394 |
| *ABCF2* | 4.904819 | *ZNF684* | -5.46139 |
| *TXNRD3* | 4.900529 | *HTR1F* | -5.46087 |
| *RAB13* | 4.898609 | *GPR22* | -5.45838 |
| *COPS5* | 4.896887 | *SORBS2* | -5.45451 |
| *CDKN1C* | 4.895454 | *ADPRHL1* | -5.45264 |
| *SH3BGRL* | 4.879347 | *TRH* | -5.45184 |
| *ATP6V0E1* | 4.875428 | *RHEBL1* | -5.44981 |
| *LMO1* | 4.874239 | *ASB2* | -5.44471 |
| *CBFA2T2* | 4.86787 | *ADRB1* | -5.44145 |
| *IL17RB* | 4.867338 | *OPTN* | -5.43749 |
| *LONP1* | 4.862704 | *EIF4B* | -5.43263 |
| *FAM149A* | 4.855261 | *KIAA1324* | -5.42515 |
| *BTNL9* | 4.855215 | *ACAD9* | -5.4205 |
| *METTL17* | 4.855199 | *MRPS30-DT* | -5.4081 |
| *ARHGEF6* | 4.852381 | *GPR63* | -5.40781 |
| *TBC1D14* | 4.849227 | *C1QL3* | -5.39788 |
| *TSPAN33* | 4.846837 | *IL7R* | -5.39146 |
| *SNX8* | 4.846147 | *KYNU* | -5.38725 |
| *CCND1* | 4.843237 | *KCNN4* | -5.37877 |
| *TAF6L* | 4.841535 | *NLK* | -5.37056 |
| *PKIB* | 4.836502 | *FBXO31* | -5.36979 |
| *TNS3* | 4.832767 | *DACH2* | -5.36244 |
| *HEBP1* | 4.830536 | *TRPV2* | -5.36166 |
| *TRAPPC1* | 4.826731 | *SLC25A25* | -5.34873 |
| *RASL10B* | 4.825732 | *SMARCA2* | -5.34546 |
| *TNFRSF14* | 4.824204 | *DUSP2* | -5.34162 |
| *ERO1B* | 4.823527 | *MAEL* | -5.33207 |
| *CCDC91* | 4.823077 | *MTX3* | -5.32481 |
| *RPS19* | 4.816319 | *MFSD6L* | -5.32217 |
| *LOC101060391* | 4.810687 | *ITGA7* | -5.31566 |
| *TMX3* | 4.809112 | *KCNJ4* | -5.30646 |
| *TNFRSF10B* | 4.80852 | *CSGALNACT1* | -5.30326 |
| *CENPM* | 4.807374 | *MMP16* | -5.30211 |
| *SYNJ2BP* | 4.803994 | *SLC7A8* | -5.29319 |
| *CADM1* | 4.801867 | *PPM1N* | -5.29041 |
| *GPX3* | 4.801622 | *NPIPB15* | -5.28806 |
| *CREG1* | 4.798102 | *KCNAB2* | -5.28618 |
| *BCL10* | 4.795231 | *KIAA1211L* | -5.28256 |
| *RARRES2* | 4.793399 | *GABRA2* | -5.27549 |
| *BTBD17* | 4.790727 | *LINC01011* | -5.27032 |
| *TRIB2* | 4.787521 | *SYT12* | -5.26796 |
| *TMEM108* | 4.787005 | *NDFIP2* | -5.265 |
| *FAM35BP* | 4.78545 | *IAH1* | -5.25548 |
| *CLMN* | 4.78131 | *TEX26* | -5.24883 |
| *TRAF3IP2* | 4.780728 | *ANKRD36B* | -5.24469 |
| *HEBP2* | 4.778838 | *KBTBD8* | -5.23565 |
| *DTNA* | 4.778369 | *SYBU* | -5.23177 |
| *TAS2R14* | 4.77748 | *SLC20A1* | -5.21654 |
| *ATP2C1* | 4.776924 | *FANCG* | -5.21415 |
| *MEGF11* | 4.771652 | *ANKRD12* | -5.21221 |
| *DOCK4* | 4.77089 | *LZTS1* | -5.2102 |
| *TRIM37* | 4.770579 | *PRMT8* | -5.20851 |
| *FAAH* | 4.76479 | *C1orf115* | -5.19937 |
| *CD99* | 4.763824 | *SLC36A1* | -5.18821 |
| *HTR7* | 4.759835 | *SEL1L3* | -5.18753 |
| *PLXNA1* | 4.759328 | *ATOH7* | -5.17759 |
| *CNOT7* | 4.756426 | *SEMA4C* | -5.17588 |
| *TRAPPC12* | 4.747774 | *AKAP5* | -5.16203 |
| *EGLN1* | 4.746602 | *AP1S2* | -5.1603 |
| *PRH2* | 4.74068 | *DGCR9* | -5.15933 |
| *KLHL32* | 4.736887 | *SDK2* | -5.15709 |
| *CLK3* | 4.735694 | *11-Sep* | -5.1477 |
| *PTPRA* | 4.727396 | *SERTAD4* | -5.13514 |
| *TMEM167A* | 4.720511 | *EMX1* | -5.12626 |
| *CXCL5* | 4.71927 | *DOC2A* | -5.12156 |
| *BEND5* | 4.716396 | *PITPNM2* | -5.11621 |
| *TCF7L1* | 4.716182 | *SLC37A4* | -5.11191 |
| *ETV6* | 4.714493 | *PPP2R3C* | -5.10707 |
| *TMX1* | 4.71367 | *CCDC149* | -5.10683 |
| *SPARC* | 4.712032 | *ZFYVE19* | -5.09956 |
| *RASGRP2* | 4.711624 | *RBMS1* | -5.08431 |
| *MAVS* | 4.711314 | *LIPC* | -5.07616 |
| *MTM1* | 4.711033 | *ECM1* | -5.07355 |
| *TOR1AIP2* | 4.708905 | *SPATA2L* | -5.07128 |
| *SPX* | 4.698735 | *SH3RF3* | -5.07056 |
| *MMP15* | 4.698721 | *A1BG-AS1* | -5.06562 |
| *C8orf33* | 4.698576 | *EXOC3L2* | -5.06357 |
| *MYRIP* | 4.698219 | *NHLRC1* | -5.06056 |
| *PPP1CC* | 4.695841 | *CHRNA7* | -5.05668 |
| *SEMA3D* | 4.695036 | *DNAH2* | -5.04672 |
| *ZNF583* | 4.689015 | *SLC39A10* | -5.03999 |
| *RB1* | 4.687162 | *ICAM5* | -5.03866 |
| *NME6* | 4.680804 | *HSD17B11* | -5.03478 |
| *GUCY1A1* | 4.678315 | *WNT10B* | -5.03323 |
| *IFIT5* | 4.678132 | *CCL28* | -5.03272 |
| *EIF4G1* | 4.673853 | *AFAP1L2* | -5.03 |
| *NEU4* | 4.663618 | *RASIP1* | -5.02928 |
| *CELF3* | 4.662484 | *CYB5B* | -5.02781 |
| *FYN* | 4.659691 | *GUSB* | -5.02579 |
| *ENHO* | 4.652005 | *NECAB2* | -5.02198 |
| *OPLAH* | 4.646116 | *HRH2* | -5.01388 |
| *SBF2* | 4.637695 | *SLIT2* | -5.00819 |
| *TMEM47* | 4.636735 | *SQLE* | -5.00053 |
| *DIRC2* | 4.636202 | *FDXR* | -4.99611 |
| *NRN1* | 4.634764 | *DSE* | -4.98993 |
| *OGFRL1* | 4.629658 | *PCDH7* | -4.98588 |
| *ANKRD6* | 4.62643 | *MCM3* | -4.97914 |
| *VSIG10L* | 4.626324 | *TNNI3* | -4.97771 |
| *AMFR* | 4.622392 | *LOC100996385* | -4.97674 |
| *SLC7A7* | 4.617561 | *RAP2B* | -4.96849 |
| *RPL21* | 4.615252 | *TEX47* | -4.96768 |
| *DENND5A* | 4.613578 | *S100A10* | -4.96331 |
| *TMEM106B* | 4.605466 | *PTGIS* | -4.96104 |
| *RPAP2* | 4.603224 | *MDFI* | -4.95786 |
| *ANKRD9* | 4.599724 | *SLCO1C1* | -4.95438 |
| *CENPB* | 4.59822 | *RMC1* | -4.94886 |
| *SLFN11* | 4.597709 | *TSPAN1* | -4.94359 |
| *ZEB1* | 4.596266 | *OLFM4* | -4.93948 |
| *SMPD1* | 4.595533 | *FIP1L1* | -4.93463 |
| *USP53* | 4.594637 | *ARPC2* | -4.93437 |
| *SURF4* | 4.593034 | *SLC25A37* | -4.93402 |
| *GSR* | 4.586994 | *CDC6* | -4.93116 |
| *SPCS1* | 4.582367 | *POC1A* | -4.92734 |
| *MRPS6* | 4.578457 | *FAM133CP* | -4.92653 |
| *PLEKHG1* | 4.578132 | *PTGER4* | -4.91952 |
| *SLC25A18* | 4.576246 | *KCNMB2* | -4.91328 |
| *ARHGEF26-AS1* | 4.570691 | *CEP72* | -4.91162 |
| *CLDN3* | 4.569692 | *LOC730098* | -4.8942 |
| *TSC22D4* | 4.567916 | *GK5* | -4.89392 |
| *NDUFB8* | 4.567526 | *TLX2* | -4.89154 |
| *PDE4D* | 4.566774 | *TCEAL9* | -4.89135 |
| *S100A16* | 4.564291 | *PRPH* | -4.88988 |
| *NAB1* | 4.559391 | *FAM129B* | -4.88351 |
| *TRIP6* | 4.546812 | *BFSP1* | -4.88351 |
| *NCK2* | 4.543412 | *KLHDC1* | -4.88065 |
| *ARL6* | 4.54271 | *THTPA* | -4.86052 |
| *BRMS1* | 4.542285 | *FILIP1L* | -4.85906 |
| *ANO5* | 4.530946 | *TNNT1* | -4.8492 |
| *GFAP* | 4.530879 | *MYO5C* | -4.84475 |
| *NFATC2IP* | 4.529766 | *SATL1* | -4.83084 |
| *SLC41A1* | 4.527152 | *B2M* | -4.82863 |
| *LOC101926935* | 4.526853 | *LOC149373* | -4.82451 |
| *PLCH2* | 4.523689 | *AP1S3* | -4.82349 |
| *NADSYN1* | 4.522908 | *SCG5* | -4.8114 |
| *AQP4* | 4.518411 | *GAS2L1P2* | -4.80907 |
| *PDPN* | 4.518377 | *LAG3* | -4.79993 |
| *H3F3C* | 4.517962 | *LMAN1L* | -4.79683 |
| *RPLP0P2* | 4.513961 | *ZNF142* | -4.78863 |
| *PSTPIP1* | 4.513959 | *PXYLP1* | -4.78397 |
| *LYPD1* | 4.512915 | *DPY19L2P4* | -4.78171 |
|  |  | *NR2C2AP* | -4.77407 |
|  |  | *HAPLN1* | -4.77075 |
|  |  | *ULK2* | -4.77035 |
|  |  | *HTR2C* | -4.76742 |
|  |  | *C19orf12* | -4.76273 |
|  |  | *MIR9-3HG* | -4.76188 |
|  |  | *SCARA5* | -4.75574 |
|  |  | *CRTAC1* | -4.75404 |
|  |  | *SPIB* | -4.75365 |
|  |  | *LRRK2* | -4.75324 |
|  |  | *MAFB* | -4.75091 |
|  |  | *ARID5A* | -4.74854 |
|  |  | *PMEPA1* | -4.74766 |
|  |  | *DIO3* | -4.74625 |
|  |  | *ISCA1* | -4.74603 |
|  |  | *OLFML2B* | -4.74018 |
|  |  | *KCNT2* | -4.73352 |
|  |  | *SYCE1* | -4.73336 |
|  |  | *MYO19* | -4.73306 |
|  |  | *GREM1* | -4.72749 |
|  |  | *ATP10A* | -4.72421 |
|  |  | *IL13RA2* | -4.71604 |
|  |  | *MAP6* | -4.71464 |
|  |  | *CCDC14* | -4.71463 |
|  |  | *RNF222* | -4.71224 |
|  |  | *ARX* | -4.69735 |
|  |  | *GPR150* | -4.6863 |
|  |  | *ZNF215* | -4.67864 |
|  |  | *COMMD2* | -4.67845 |
|  |  | *MEI1* | -4.67325 |
|  |  | *LOC101926934* | -4.671 |
|  |  | *ZACN* | -4.66719 |
|  |  | *RFC3* | -4.66282 |
|  |  | *GPR83* | -4.66193 |
|  |  | *ACE* | -4.65658 |
|  |  | *IKBIP* | -4.65432 |
|  |  | *EFNB1* | -4.65232 |
|  |  | *GASAL1* | -4.6508 |
|  |  | *WFIKKN1* | -4.64937 |
|  |  | *DCLK1* | -4.64599 |
|  |  | *SFMBT2* | -4.64505 |
|  |  | *FBLN5* | -4.64362 |
|  |  | *MAPK9* | -4.6434 |
|  |  | *B3GNT2* | -4.64175 |
|  |  | *SNTB2* | -4.63792 |
|  |  | *LINC00599* | -4.63396 |
|  |  | *PGAP2* | -4.6331 |
|  |  | *TMSB15B* | -4.63051 |
|  |  | *TULP4* | -4.63015 |
|  |  | *MKX* | -4.62983 |
|  |  | *NIT2* | -4.62799 |
|  |  | *DNAJC22* | -4.62533 |
|  |  | *SLC9A5* | -4.62432 |
|  |  | *FAM228A* | -4.61598 |
|  |  | *PRSS1* | -4.61384 |
|  |  | *GALNT2* | -4.61112 |
|  |  | *PRPH2* | -4.60902 |
|  |  | *C3orf80* | -4.60808 |
|  |  | *CNNM1* | -4.60128 |
|  |  | *SCG2* | -4.59866 |
|  |  | *BAHD1* | -4.59732 |
|  |  | *RBFOX3* | -4.59565 |
|  |  | *MYO15A* | -4.59366 |
|  |  | *C20orf141* | -4.59225 |
|  |  | *KNG1* | -4.59186 |
|  |  | *RBM24* | -4.58508 |
|  |  | *KIAA1107* | -4.58398 |
|  |  | *LOC284454* | -4.58126 |
|  |  | *SLA* | -4.57985 |
|  |  | *SMIM24* | -4.5794 |
|  |  | *PDZD4* | -4.57626 |
|  |  | *VCL* | -4.57543 |
|  |  | *SPARCL1* | -4.57465 |
|  |  | *P3H2* | -4.56783 |
|  |  | *GDAP1* | -4.5678 |
|  |  | *STXBP5* | -4.56734 |
|  |  | *TLL1* | -4.56525 |
|  |  | *ARC* | -4.56388 |
|  |  | *ARPC3* | -4.56242 |
|  |  | *SLC16A8* | -4.56131 |
|  |  | *NDUFB6* | -4.56082 |
|  |  | *UBR7* | -4.55859 |
|  |  | *AMZ2P1* | -4.54839 |
|  |  | *PGAP3* | -4.54363 |
|  |  | *BICC1* | -4.54122 |
|  |  | *MAPK6* | -4.54048 |
|  |  | *SWAP70* | -4.53812 |
|  |  | *LOXL1* | -4.53765 |
|  |  | *ZNF436* | -4.52224 |
|  |  | *RSPH9* | -4.51721 |

Abbreviations: PLS2, the second component in the partial least squares.

Table S7. Gene-category enrichment analysis of the significant PLS2- gene sets.

| **Biological process terms** |
| --- |
| Prostaglandin biosynthetic process |
| Positive regulation of protein phosphorylation |
| Apoptotic process |
| Cell adhesion |
| Spermatogenesis |
| Embryo implantation |
| Male gonad development |
| Response to mechanical stimulus |
| Response to fructose |
| Response to manganese ion |
| Response to lithium ion |
| Positive regulation of vascular endothelial growth factor production |
| Regulation of collagen catabolic process |
| Negative regulation of extracellular matrix disassembly |
| Cyclooxygenase pathway |
| Lipoxygenase pathway |
| Bone mineralization |
| Ovulation |
| Positive regulation of prostaglandin biosynthetic process |
| Positive regulation of synaptic plasticity |
| Negative regulation of synaptic transmission, dopaminergic |
| Response to estradiol |
| Positive regulation of peptidyl-serine phosphorylation |
| Response to vitamin D |
| NAD biosynthesis via nicotinamide riboside salvage pathway |
| Response to tumor necrosis factor |
| Cellular response to UV |
| Intracellular signal transduction |
| Maintenance of permeability of blood-brain barrier |
| Positive regulation of protein import into nucleus |
| Long-chain fatty acid biosynthetic process |
| Positive regulation of nitric oxide biosynthetic process |
| Negative regulation of cell cycle |
| Negative regulation of smooth muscle contraction |
| Positive regulation of smooth muscle contraction |
| Decidualization |
| Platelet-derived growth factor receptor signaling pathway |
| Positive regulation of fibroblast proliferation |
| Regulation of inflammatory response |
| Proteolysis involved in cellular protein catabolic process |
| Regulation of fibrinolysis |
| Negative regulation of calcium ion transport |
| Positive regulation of synaptic transmission, glutamatergic |
| Palate development |
| Response to fatty acid |
| Positive regulation of cell cycle arrest |
| Cellular response to lead ion |
| Cellular response to ATP |
| Cellular response to hypoxia |
| Cellular response to non-ionic osmotic stress |
| Positive regulation of transforming growth factor beta production |
| Mitotic cell cycle arrest |
| Positive regulation of fibroblast growth factor production |
| Positive regulation of brown fat cell differentiation |
| Positive regulation of platelet-derived growth factor production |
| Melanocyte proliferation |
| Negative regulation of intrinsic apoptotic signaling pathway in response to osmotic stress |
| Melanocyte apoptotic process |
| Negative regulation of extracellular matrix organization |
| Neuron projection extension |
| Response to angiotensin |

Abbreviations: PLS, partial least squares.

**Supplementary Figures**


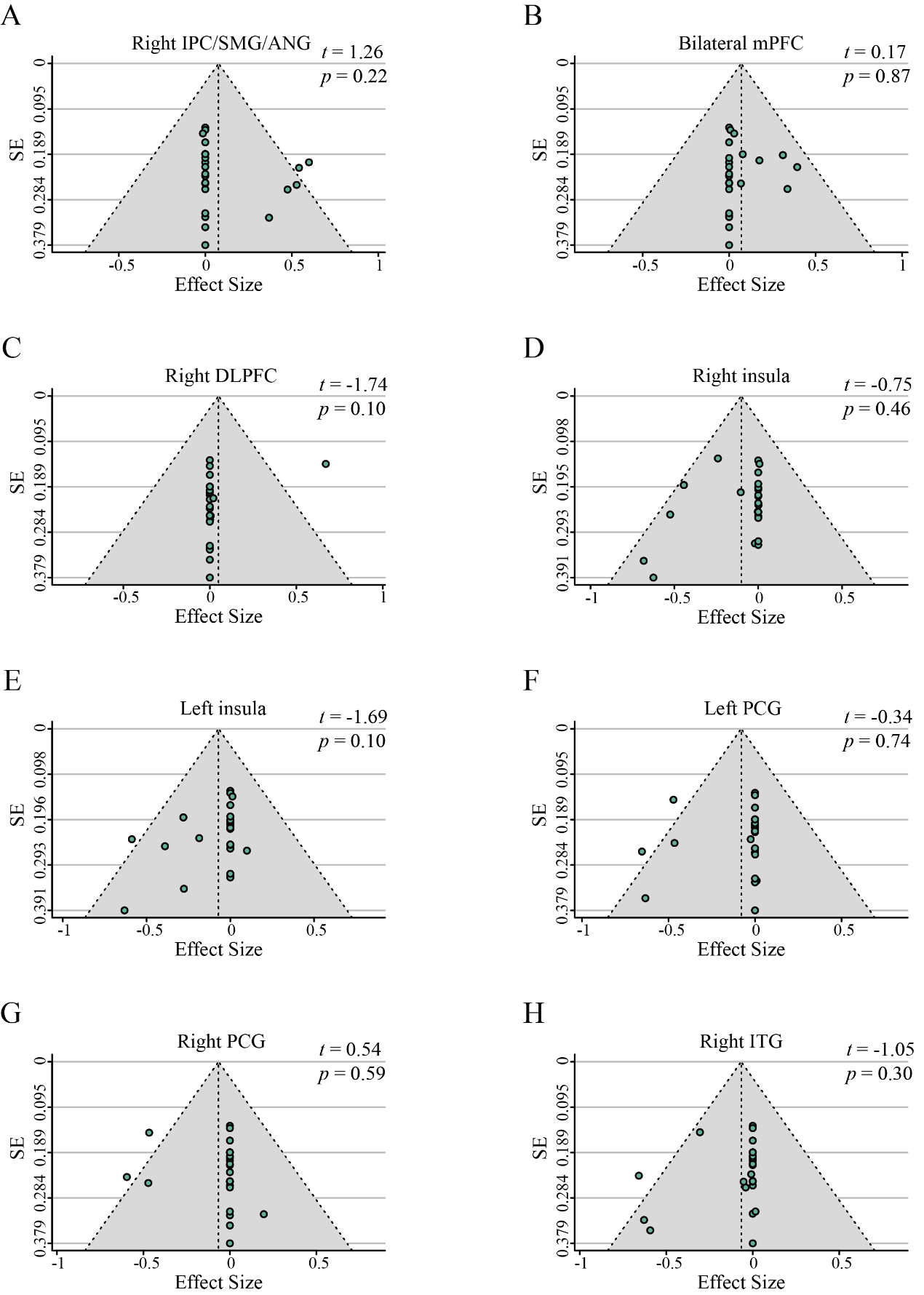


**Fig. S1.** Funnel plots of regions with significantly altered functional network centrality. Each dataset is marked as a dot. Abbreviations: ANG, angular gyrus; DLPFC, dorsolateral prefrontal cortex; IPC, inferior parietal cortex; ITG, inferior temporal gyrus; mPFC, medial prefrontal cortex; PCG, postcentral gyrus; SE, standard error; SMG, supramarginal gyrus.

**
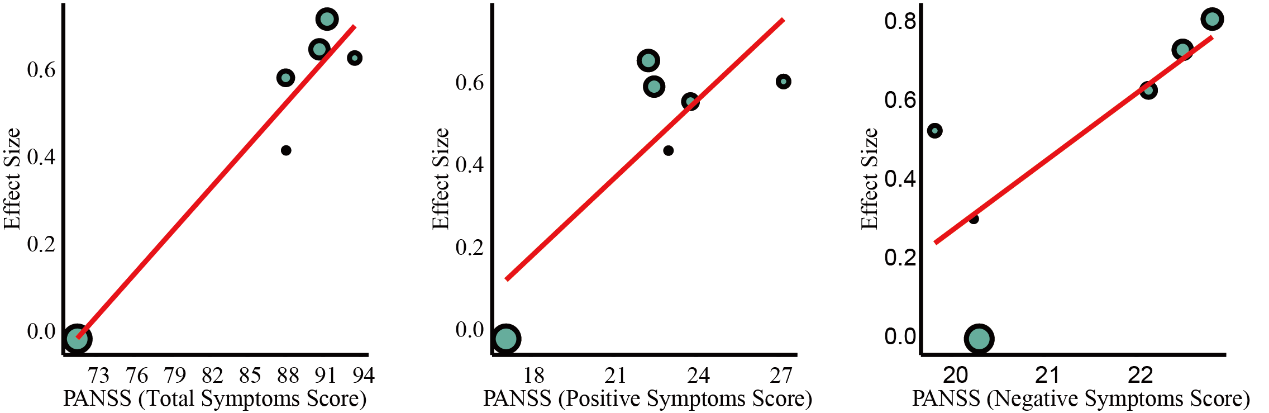
**

**Fig. S2.** Scatter plots of the relationship between PANSS scores (total, positive, and negative) and altered FNC in the right IPC after removing studies with null effect sizes from the meta-analysis. Abbreviations: PANSS, Positive and Negative Syndrome Scale.


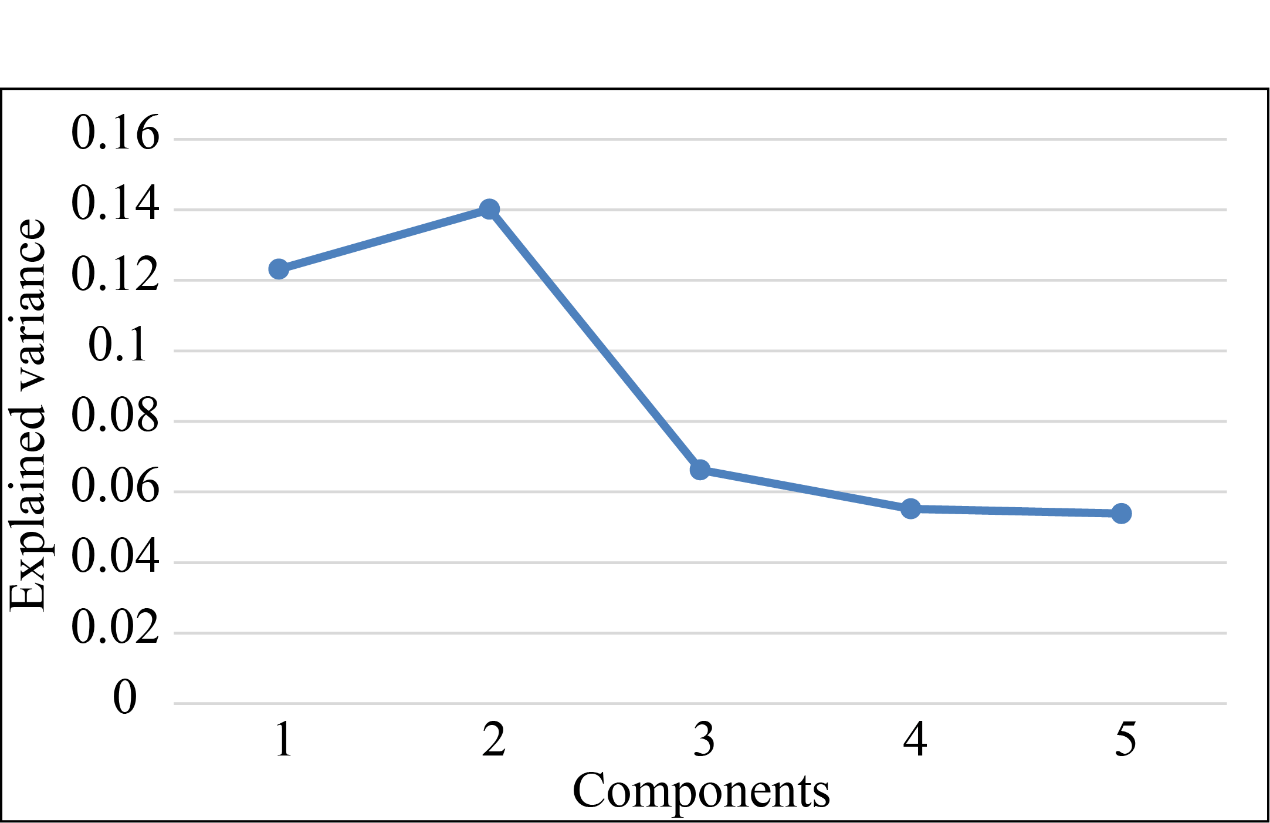


**Fig. S3.** Explained variance for the top five components from the PLS regression.
